# Origin of the Dengue Fever Mosquito, *Aedes aegypti*, in California

**DOI:** 10.1371/journal.pntd.0003029

**Published:** 2014-07-31

**Authors:** Andrea Gloria-Soria, Julia E. Brown, Vicki Kramer, Melissa Hardstone Yoshimizu, Jeffrey R. Powell

**Affiliations:** 1 Department of Ecology and Evolutionary Biology, Yale University, New Haven, Connecticut, United States of America; 2 California Department of Public Health, Vector-Borne Disease Section, Sacramento, California, United States of America; The Kenya Medical Research Institute (KEMRI), Kenya

## Abstract

Dengue fever is among the most widespread vector-borne infectious diseases. The primary vector of dengue is the *Aedes aegypti* mosquito. *Ae. aegypti* is prevalent in the tropics and sub-tropics and is closely associated with human habitats outside its native range of Africa. While long established in the southeastern United States of America where dengue is re-emerging, breeding populations have never been reported from California until the summer of 2013. Using 12 highly variable microsatellite loci and a database of reference populations, we have determined that the likely source of the California introduction is the southeastern United States, ruling out introductions from abroad, from the geographically closer Arizona or northern Mexico populations, or an accidental release from a research laboratory. The power to identify the origin of new introductions of invasive vectors of human disease relies heavily on the availability of a panel of reference populations. Our work demonstrates the importance of generating extensive reference databases of genetically fingerprinted human-disease vector populations to aid public health efforts to prevent the introduction and spread of vector-borne diseases.

## Author Summary

Dengue fever is a vector-borne disease that has dramatically increased in frequency and severity in recent decades, affecting over half of the world's population. The primary vector of dengue is *Aedes aegypti*. Breeding populations of this mosquito were reported for the first time in California in summer 2013, and the origin of these populations was unknown. We genotyped *Ae. aegypti* from California and compared their genetic composition and diversity against reference populations from Asia and the Americas, and with laboratory strains, to identify the source of this introduction. We identified the southeastern United States of America as the likeliest origin of California *Ae. aegypti*. This information will aid the design of containment, control, and elimination strategies to prevent the establishment of a permanent breeding vector population in the state. Note that the resolution achieved in tracking the origin of new disease-vector introductions is proportional to the completeness of the reference dataset. Our analysis was delayed to gather further data on regions close to California and would otherwise have been completed within a week. This work highlights the importance of having an extended dataset of human disease-vector genotypes in place prior to the occurrence of invasions that compromise public health.

## Introduction

**Figure 1:**
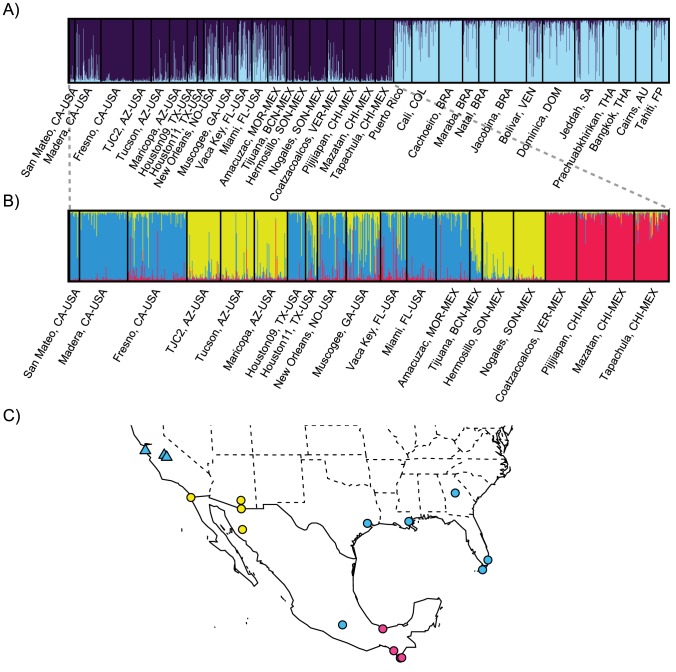
Genetic structure within pantropical populations of *Aedes aegypti*. STRUCTURE bar plots indicating relatedness of *Aedes aegypti* populations based on 12 microsatellite loci. Each vertical bar represents an individual. The height of each bar represents the probability of assignment to each of K optimal clusters (different colors) determined using the Delta K method. (**A**) North America and Asian populations (K = 2), and (**B**) North American populations (K = 3). (**C**) Map indicating the North American geographic locations sampled in this study. (**Δ**) California, (○) other locations in North America.

Dengue fever is re-emerging in the United States of America (USA) after many years of absence [1], a trend also observed around the world [2]. Risk for dengue infection coincides with the distribution of mosquitoes capable of transmitting dengue virus (DENV). In most areas of the world, *Aedes aegypti* is the mosquito species responsible for DENV transmission. *Ae. aegypti* is a domestic species in the sense that, outside Africa, it is closely associated with human habitats and is often transported by humans and their commerce [3]. In the USA, *Ae. aegypti* is currently found in the southern states, Arizona eastward, with year-round breeding confined to latitudes below 33°N [4]. The state of California has an active and extensive mosquito-monitoring program since 1917 and in the past has only detected sporadic specimens of *Ae. aegypti* near airports [5]. Confirmed breeding populations of *Ae. aegypti* in California were never reported until the summer of 2013, when they were found in the Central Valley counties of Fresno and Madera and the coastal county of San Mateo (Figure 1C). When exotic vectors of important diseases are introduced into a new and densely populated region like California, it is important to understand where they originated in order to implement appropriate containment, control, and elimination strategies. As part of our ongoing studies of the genetic variation of *Ae. aegypti* populations, we report here the use of 12 highly polymorphic microsatellites and a database of reference populations to identify the likely origin of the California *Ae. aegypti* detected in 2013. The power to identify the source of the introduction relies heavily on the completeness of the reference panel and thus, the origin could be further narrowed down as the reference database gets expanded.

## Methods

### *Aedes aegypti* collections

*Aedes aegypti* adults and larvae for this study came from collections made between 2004 and 2013 from 33 locations across 11 countries in the Americas and Asia (Table S1). Mosquitoes that were genotyped arrived as either eggs from oviposition traps, or as larvae or adults in 70–100% ethanol. Eggs were hatched at the Yale School of Epidemiology and Public Health insectary and reared to adults for identification and preservation in 100% ethanol at −20°C. Mosquitoes included in this study came directly from the field, from multiple oviposition traps, unless indicated in Table S1.

### DNA extraction and genotyping

Total nucleic acids were extracted from 1205 individual *Aedes aegypti* mosquitoes with the DNeasy Blood and Tissue kit (Qiagen), according to manufacturer instructions. Samples were treated with 4 µl of RNAse A (Qiagen) and stored at −20°C until further analysis. Our reference dataset included eleven populations of *Ae. aegypti* from around the world that have been screened for allele frequencies at 12 highly polymorphic microsatellite loci in previous studies [6], [7]. Additionally, we genotyped 19 natural populations from Asia and the Americas as well as samples of 3 populations from California: Madera, Fresno, and San Mateo; for the same 12 loci (Table S1). Individual mosquitoes were genotyped as described in Brown et al. [6]. The microsatellite loci analyzed were: A1, AC2, CT2, AG2, B2, B3, A9, AC4, AC1, AC5, AG1, and AG4 (Table S2). Briefly, polymerase chain reactions were conducted as 10 µl reactions using the Type-it Microsatellite PCR Master Mix (Qiagen), 25 nM of each forward primer, 250 nM of each reverse primer, and 500 nM of a fluorescently labeled M13 primer. Thermocycler conditions were as follows: 94°C×10′, 35×(94°C×30″, 54°C×30″, 72°C×30″), and 72°C×5′. The resulting products were processed for fragment analysis at the DNA Analysis Facility at Science Hill at Yale University, using GS 500 Rox internal size standard (Applied Biosystems). Microsatellite alleles were scored using GeneMapper v4.0 (Applied Biosystems).

### Analyses

All microsatellite loci were analyzed for within-population deviations from Hardy-Weinberg equilibrium (HWE) by F_is_, as well as for linkage disequilibrium (LD) among loci pairs, using the online version of the GENEPOP software [8], [9] with 10,000 dememorizations, 1,000 batches, and 10,000 iterations per batch for both tests. The G_is_, analogue to F_is_, was also calculated in GenoDive v.2.0b25 [10] to evaluate deviations from HWE using 10,000 permutations. A Bonferroni correction was applied to the resulting matrix to correct for multiple testing. The Adegenet package v. 1.3.9. [11] available for the R software v. 3.0.1. [12] was used to compute the number of alleles at each microsatellite loci and the average observed and expected heterozygosities per population. Principal Component Analysis was implemented with the same package. Allelic richness and private allelic richness were calculated in HPRARE [13], which uses rarefaction to correct for unequal sample sizes.

Population structure and assignments of individuals from the three California populations to their genetic cluster of origin was performed via the Bayesian clustering method implemented by the software STRUCTURE v. 2.3 [14]. STRUCTURE identifies genetic clusters and assigns individuals to these clusters with no *a priori* information of sample location. The most likely number of clusters (K) was determined by conducting 20 independent runs from K = 1 to 29 on individuals of all American and Asian populations, and from K = 1 to 21 on individuals of the North American cluster. Each run assumed an admixture model and correlated allele frequencies using a burn-in value of 100,000 iterations followed by 500,000 repetitions. The optimal number of K clusters was determined following the guidelines of Prichard et al. [14] and the Delta K method from Evanno et al. [15], [16]. Results were plotted with the program DISTRUCT v.1.1 [17].

GENECLASS2 [18] was used to perform individual and group assignment tests on the three California populations against the reference population dataset using the Bayesian criteria for likelihood estimation [19]. The reference dataset included all populations identified in STRUCTURE as belonging to the same genetic cluster as the California locations. The assignment tests allowing computation of probabilities were run using Monte-Carlo resampling with *N = 10,000* and *α = 0.05* [20]. Results from the individual probability assignment tests were compiled and plotted using the software R v. 3.0.1. [12]. Additionally, individual assignment tests of 5 individuals from each of the reference populations and group self-assignment tests were performed to evaluate the accuracy of the assignment method. Group self-assignment tests were 100% accurate for all reference populations and genetic clusters. The individual approach considering the probabilities of computation assigned the highest probability to the correct population of origin for 64.5% of the individuals, while the correct genetic cluster was that of the highest probability for 90.6% of the individuals.

To test for possible sample size effects on STRUCTURE, we evaluated the possibility that uneven sample size might have an effect on the STRUCTURE analysis and our conclusions. We performed resampling of the data from all 20 North American samples, with and without replacement, with all samples equal to 15 (without replacement) and 30 (with replacement) genotypes. When the genotypes were subsampled without replacement, 6 out of 10 runs resulted in K = 3 being the optimal number of clusters, while the remaining 4 had an optimal K = 2. When the genotypes were resampled with replacement, the optimal number of clusters was K = 3 for 7 out of 10 runs, K = 5 for 2 out of 10 runs, and K = 2 for 1 out of 10 runs. In all simulations, the California samples had greatest affinity to the Southeast US group samples at K = 3 (data not shown).

Raw data (allele frequencies) can be found in Supplementary Materials, Table S4.

## Results

### Genetic diversity in *Ae. aegypti* from California is similar to that in natural populations

Allele richness and heterozygosity values were estimated for both laboratory colonies and natural populations of *Ae. aegypti*, to address whether the California introduction was a consequence of a laboratory release (Table 1 and S3). The mean observed average heterozygosity (H_o_) for natural populations was significantly higher than that of laboratory strains (0.5153±0.076 and 0.3015±0.184 respectively; P<0.001). Allelic richness across loci within natural strains was 3.6206±0.5681 and 2.1633±0.6104 within lab strains, consistent with the genetic diversity pattern observed from the heterozygosity values. When the California populations were analyzed independently, the H_o_ values of all three California collections fell within the values of the natural populations (San Mateo: 0.4635; Madera: 0.5563; and Fresno: 0.4904; P = 0.7052; Table 1 and S3).

**Table 1:** Genetic diversity of *Aedes aegypti* populations.

Locality	H_o_	H_e_	AR
San Mateo	0.4635	0.4949	3.65
Madera	0.5563	0.5435	3.53
Fresno	0.4904	0.5254	3.67
Pantropical* ± SD	0.5153±0.0760	0.5166±0.0764	3.6206±0.5681
Lab strains** ± SD	0.3015±0.1840	0.3052±0.1963	2.1633±0.6104

H_o_ = observed heterozygosity; H_e_ = expected heterozygosity; AR = Allelic richness estimated by rarefaction (N = 30 genes).

*Pantropical = mean across populations from Asia and the Americas.

**Lab strains = mean across Hamburg, Rockefeller, and Liverpool laboratory strains provided by David Severson (University of Notre Dame, Indiana).

### California *Ae. aegypti* is closely related to populations in the southeastern USA

To identify the potential source of the California introduction, we analyzed the pattern of population structure in a dataset that included the three California populations (Madera, Fresno, and San Mateo Counties) and a set of reference populations from the Americas and Asia (Table S1). Because California populations belong to the pantropical unit outside Africa known as *Ae. aegypti aegypti* [6], Fig. S1, in our comparisons we excluded Africa as a possible origin. Bayesian clustering analysis on these collections identified two main clusters (Δ K = 2) that split North America (USA and Mexico) from a group comprising the Caribbean, South America, and Asia (Figure 1A). Some admixture was observed between the groups, especially within the Florida populations. An independent Bayesian clustering analysis on the North American populations identified three clusters (Δ K = 3; Figure 1B), corresponding with 1) Southern Mexico, 2) Arizona and Northern Mexico, and 3) southeastern USA, California, and the population in central Mexico (Amacuzac, Morelos). Therefore, California populations are genetically distinct from *Ae. aegypti* from Asia, Australia, Tahiti, Hawaii, and South America and thus these locales are highly unlikely to have been the source of the invasion. Interestingly, *Ae. aegypti* from California is most closely related to USA populations east of Arizona, rather than to Arizona populations, the state immediately adjacent to California (Figure 1B). Geographic clustering can be observed in Figure 1C when we exclude the California populations.

**Figure 2:**
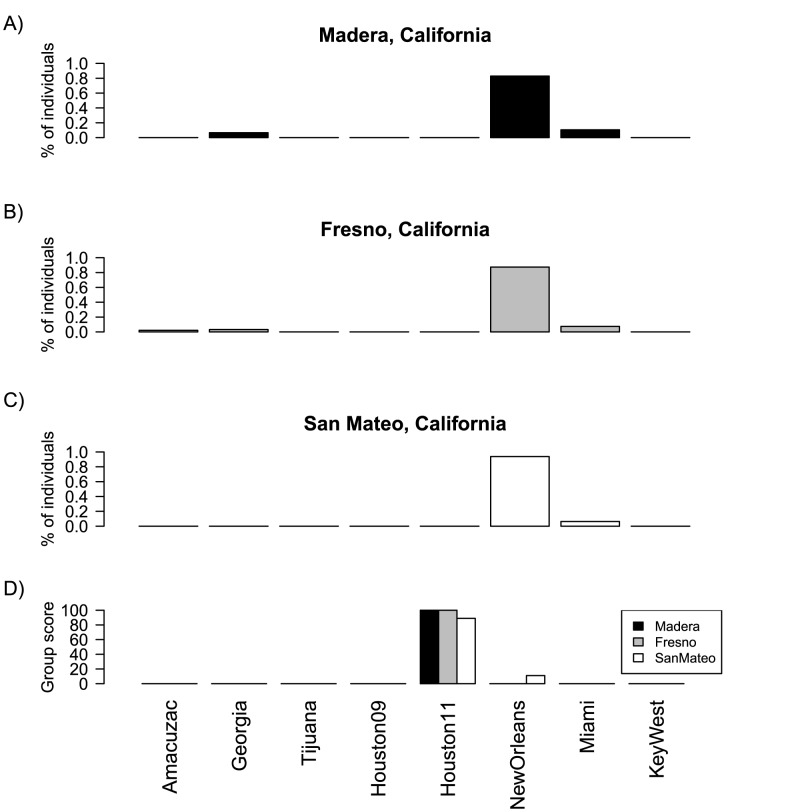
Individual and group mosquito genetic assignments. Percentage of individuals from Madera (**A**), Fresno (**B**), and San Mateo (**C**) counties assigned with the highest probability to each of the reference populations. (**D**) Scores calculated for each of the reference populations after group assignment of each of three California populations. Assignments were performed using Bayesian criteria for likelihood estimation with GENECLASS 2.0.

Likelihood estimation methods [18] used to more finely detail the origin of the California introduction, consistently assigned California individuals to New Orleans with the highest probability (individual assignment tests; Fig. 2A–C); or California populations to Houston, with New Orleans the second most probable source (group assignment tests; Fig. 2D).

In addition, we ran a Principal Components Analysis (PCA) on the allele frequencies of the 20 North American samples. The California samples are indistinguishable from samples from the Southeast US, but distinct from all other samples (Figure S2).

## Discussion

Our analysis suggests that *Ae. aegypti* was introduced to California from the southeastern USA by multiple founder mosquitoes. Populations of recently introduced species are often relatively low in genetic variation due to the new populations having been started by a few founders. In comparing the level of genetic variation of California *Ae. aegypti* populations to other populations there is no indication of a decrease in genetic diversity (Table 1 and S3), including no decrease in the numbers of alleles at loci, the genetic parameter most sensitive to founder effects. The high level of genetic diversity observed in the California populations also argues against the release of a laboratory strain (Table 1 and S3). Additionally, California laboratories maintaining *Ae. aegypti* indicated that no recent strains of *Ae. aegypti* from the southeast USA are being currently reared in California, thus eliminating this alternative hypothesis.

It is conceivable that the California populations are not recent introductions, which might also explain the relatively high level of genetic variation. We consider this less likely due to the fact that *Ae. aegypti* are morphologically quite distinctive and would have been detected given the extent of mosquito monitoring activities in California. The fact that sporadic *Ae. aegypti* in or near airports in California have been previously reported indicates that the quality and intensity of monitoring has been sufficient to detect this species.

Our data indicates that the origin of the California invasion can be tracked to the region of New Orleans and Houston, cities ∼300 km apart, and likely did not originate from spillover from geographically adjacent regions that have long had *Ae. aegypti* (Arizona and northern Mexico). This suggests that commerce via air, railroad, or trucks from the New Orleans/Houston areas may have been responsible, though the mode of introduction remains unknown.

The extensive mosquito surveillance program in California allowed for a rapid response to the initial detections, emphasizing the importance of enhanced surveillance and personnel training, especially in regions susceptible to invasion of exotic disease vectors. In the USA, dengue became a nationally notifiable infectious disease in 2010, and for 2013, California has reported 124 imported cases [21]. In contrast to our previous study of an *Ae. aegypti* invasion into temperate Netherlands [22], it is conceivable that the climate of California may allow for these newly detected populations to overwinter and become established. The combination of imported dengue cases and the permanent presence of the primary vector of dengue in California could eventually lead to local transmission of the disease, thus becoming an important threat to public health. Identifying the origin of the California introduction should facilitate the implementation of strategies to reduce or eliminate the movement of new vectors into the area.

The degree of accuracy in the identification of the source population of the California *Ae. aegypti* is proportional to the completeness of the reference population panel. Prior to this study, we had very sparse sampling from Arizona and northern Mexico; this delayed our analyses by several months while additional samples from this region were obtained and genotyped. Had our genetic panel been more complete, the study could have been carried out within a week. This highlights the importance of having in place extensive population genetic databases for invasive vectors prior to new invasions, which occur on a regular basis and are expected to increase as the climate patterns change. While spread of invasive vectors usually occurs inadvertently, purposeful introductions by bioterror activities could be tracked in a similar manner. An analogy can be made with the notorious 2001 anthrax event, whereby the origin of the strain was quickly traced thanks to an extensive database of DNA sequences of hundreds of anthrax genotypes [23]. More extensive reference databases of genetically fingerprinted human disease vector populations would greatly aid public health efforts to prevent the introduction and spread of vector-borne diseases.

## Supporting Information

Figure S1 California populations belong to the pantropical cluster of *Aedes aegypti*. STRUCTURE bar plots indicating relatedness of *Aedes aegypti* populations worldwide, based on 12 microsatellite loci. Each vertical bar represents an individual. The height of each bar represents the probability of assignment to K = 2 optimal clusters (different colors) determined using the Delta K method. Pantropical cluster is shown in pink and the African cluster in blue. Populations from the African cluster were originally analyzed in Brown et al. 2011 [6]. USA: United States of America, MEX: Mexico, COL: Colombia, BRA: Brazil, SA: Saudi Arabia, THA: Thailand, AU: Australia, CM: Cameroon, UG: Uganda, and SN: Senegal.
(EPS)

Figure S2 Principal Component Analysis in 20 North American samples of *Aedes aegypti* using 12 microsatellite loci. Note that California samples (*) are indistinguishable from samples from the Southeast US.
(EPS)

Table S1 Collection information for *Aedes aegypti* populations analyzed for 12 microsatellite loci.
(DOCX)

Table S2 Number of alleles per microsatellite locus.
(DOCX)

Table S3 Genetic diversity of *Aedes aegypti* populations.
(DOCX)

Table S4 Raw allele frequencies at 12 microsatellite loci.
(DOCX)

Text S1 Marker validation.
(DOCX)
