# Supplementary material for: Origin of the Dengue Fever Mosquito, Aedes aegypti, in California
Source: PLoS Negl Trop Dis. 2014 Jul 31;8(7):e3029. doi: 10.1371/journal.pntd.0003029 (PMC4117443; doi:10.1371/journal.pntd.0003029)
Supplement: Table S1 — Collection information for Aedes aegypti populations analyzed for 12 microsatellite loci. (DOCX) [file pntd.0003029.s003.docx]

Table S1. Collection information for *Aedes aegypti* populations analyzed for 12 microsatellite loci.

| Collection site* | Year of collection | *N* | Gen. in lab | Genotyping |
| --- | --- | --- | --- | --- |
| San Mateo County, California, USA  Madera, California, USA  Fresno, California, USA  Tijuana, Baja California Norte, MEX  Hermosillo, Sonora, MEX  Nogales, Sonora, MEX  Tucson (TJC2), Arizona, USA  Tucson, Arizona, USA  Maricopa County, Arizona, USA  Houston, Texas, USA  Houston, Texas, USA  New Orleans, New Orleans, USA  Muscogee County, Georgia, USA  Vaca Key, Florida, USA  Miami, Florida, USA  Puerto Rico  Amacuzac, Morelos, MEX  Coatzacoalcos, Veracruz, MEX  Pijijiapan, Chiapas, MEX  Mazatan, Chiapas, MEX  Tapachula, Chiapas, MEX  Cali, COL  Cachoeiro, BRA  Maraba, BRA  Natal, BRA  Jacobina, BRA  Bolivar, VEN  Dominica, DOM  Jeddah, SA  Prachuabkhirikan, THA  Bangkok, THA  Cairns, AU  Tahiti, FP | 2013  2013  2013  2013  2013  2013  2012  2012  2013  2009  2011  2011  2012  2009  2011  2012  2012  2008  2006  2012  2012  2013  2010  2010  2010  2013  2004  2009  2012  2009  2011  2009  2010 | 16  77  95  20  50  51  54  54  53  29  19  46  55  42  47  54  54  50  48  45  54  80  70  48  47  94  48  95  84  47  49  48  48 | 0  0  0  0  0  0  0  2  0  0  0  0  0  0  0  0  0  0  1  1  1  0  1-2  0  0  0  2  0  0  2  0  0  1 | this study  this study  this study  this study  this study  this study  this study  this study  this study  (*6*)  this study  this study  this study  (*16*)  (*10*)  this study  this study  (*6*)  (*6*)  this study  this study  this study  (*7*)  this study  this study  this study  (*6*)  (*6*)  this study  (*6*)  this study  (*6*)  (*6*) |

* USA: United States of America, MEX: Mexico, COL: Colombia, BRA: Brazil, VEN: Venezuela, DOM: Dominica, SA: Saudi Arabia, THA: Thailand, AU: Australia, and FP: French Polynesia.
