# Supplementary material for: Origin of the Dengue Fever Mosquito, Aedes aegypti, in California
Source: PLoS Negl Trop Dis. 2014 Jul 31;8(7):e3029. doi: 10.1371/journal.pntd.0003029 (PMC4117443; doi:10.1371/journal.pntd.0003029)
Supplement: Table S2 — Number of alleles per microsatellite locus. (DOCX) [file pntd.0003029.s004.docx]

Table S2. Number of alleles per microsatellite locus.

| **Loci** | **No. of alleles** |
| --- | --- |
| AC1 | 8 |
| AC2 | 6 |
| AC4 | 4 |
| AC5 | 20 |
| CT2 | 7 |
| AG1 | 7 |
| AG2 | 29 |
| AG5 | 11 |
| A1 | 10 |
| A9 | 8 |
| B2 | 5 |
| B3 | 7 |
