# Supplementary material for: Origin of the Dengue Fever Mosquito, Aedes aegypti, in California
Source: PLoS Negl Trop Dis. 2014 Jul 31;8(7):e3029. doi: 10.1371/journal.pntd.0003029 (PMC4117443; doi:10.1371/journal.pntd.0003029)
Supplement: Table S3 — Genetic diversity of Aedes aegypti populations. (DOCX) [file pntd.0003029.s005.docx]

Table S3. Genetic diversity of *Aedes aegypti* populations.

| **Locality** | **H_o_** | **H_e_** | **AR*^a^*** | **PAR*^a^*** |
| --- | --- | --- | --- | --- |
| San Mateo County, California, USA | 0.4635 | 0.4949 | 3.65 | 0.00 |
| Madera, California, USA | 0.5563 | 0.5435 | 3.53 | 0.00 |
| Fresno, California, USA | 0.4904 | 0.5254 | 3.67 | 0.01 |
| Tijuana, Baja California Norte, MEX | 0.6250 | 0.5497 | 2.91 | 0.00 |
| Hermosillo, Sonora, MEX | 0.5400 | 0.5613 | 3.81 | 0.03 |
| Nogales, Sonora, MEX | 0.5490 | 0.5585 | 3.75 | 0.04 |
| Tucson (TJC2), Arizona, USA | 0.6019 | 0.5659 | 3.69 | 0.04 |
| Tucson, Arizona, USA | 0.5478 | 0.5889 | 3.94 | 0.00 |
| Maricopa County, Arizona, USA | 0.5566 | 0.5573 | 3.87 | 0.00 |
| Houston, Texas, USA (2009) | 0.5417 | 0.4338 | 3.02 | 0.04 |
| Houston, Texas, USA (2011) | 0.4386 | 0.4159 | 2.99 | 0.02 |
| New Orleans, New Orleans, USA | 0.4386 | 0.4159 | 4.44 | 0.05 |
| Muscogee County, Georgia, USA | 0.6424 | 0.6367 | 4.35 | 0.09 |
| Vaca Key, Florida, USA | 0.5119 | 0.5891 | 4.20 | 0.13 |
| Miami, Florida, USA | 0.6667 | 0.6310 | 4.56 | 0.07 |
| Puerto Rico | 0.6000 | 0.5962 | 4.34 | 0.07 |
| Amacuzac, Morelos, MEX | 0.5401 | 0.5457 | 3.76 | 0.13 |
| Coatzacoalcos, Veracruz, MEX | 0.4183 | 0.3408 | 2.48 | 0.00 |
| Pijijiapan, Chiapas, MEX | 0.3901 | 0.4585 | 3.11 | 0.00 |
| Mazatan, Chiapas, MEX | 0.5111 | 0.4739 | 3.11 | 0.00 |
| Tapachula, Chiapas, MEX | 0.5015 | 0.4985 | 3.31 | 0.00 |
| Cali, COL | 0.4500 | 0.5023 | 3.40 | 0.06 |
| Cachoeiro, BRA | 0.4405 | 0.4788 | 3.56 | 0.04 |
| Maraba, BRA | 0.5226 | 0.5543 | 3.62 | 0.00 |
| Natal, BRA | 0.5053 | 0.4977 | 3.36 | 0.05 |
| Jacobina, BRA | 0.4264 | 0.4537 | 3.34 | 0.06 |
| Bolivar, VEN | 0.5226 | 0.5469 | 3.76 | 0.04 |
| Dominica, DOM | 0.4316 | 0.4539 | 3.22 | 0.08 |
| Jeddah, SA | 0.5913 | 0.6007 | 4.60 | 0.18 |
| Prachuabkhirikan, THA | 0.5195 | 0.5848 | 4.16 | 0.20 |
| Bangkok, THA | 0.4082 | 0.3931 | 2.53 | 0.00 |
| Cairns, AU | 0.6354 | 0.5523 | 3.43 | 0.18 |
| Tahiti, FP | 0.5764 | 0.5877 | 4.00 | 0.06 |
| PANTROPICAL* ± SD | 0.5153 ± 0.0760 | 0.5166 ± 0.0764 | 3.6206 ± 0.5681 | 0.0553 ±  0.0576 |
| **Laboratory strains** |  |  |  |  |
| Hamburg (N=54) | 0.4505 | 0.4664 | 2.62 | 0.03 |
| Rockefeller (N=54) | 0.3581 | 0.3627 | 2.40 | 0.08 |
| Liverpool (N=53) | 0.0959 | 0.0866 | 1.47 | 0.00 |
| LABORATORY STRAINS** ± SD | 0.3015 ± 0.1840 | 0.3052 ± 0.1963 | 2.1633 ± 0.6104 | 0.0367 ±  0.0404 |

H_o_ = observed heterozygocity; H_e_ = expected heterozygocity; AR = Allelic richness estimated by rarefaction (N=30 genes).

*^a^*corrected for a minimum sample size of 30 genes.

^*^Pantropical = mean across populations from Asia and the Americas.

^**^Laboratory strains = mean across Hamburg, Rockefeller, and Liverpool laboratory strains provided by David Severson (University of Notre Dame, Indiana).
