# Supplementary material for: Origin of the Dengue Fever Mosquito, Aedes aegypti, in California
Source: PLoS Negl Trop Dis. 2014 Jul 31;8(7):e3029. doi: 10.1371/journal.pntd.0003029 (PMC4117443; doi:10.1371/journal.pntd.0003029)
Supplement: Table S4 — Raw allele frequencies at 12 microsatellite loci. (DOCX) [file pntd.0003029.s006.docx]

**Table S4.** Raw allele frequencies at 12 microsatellite loci. Allele designations as in Brown et al. 2011.

|  | **AC1 locus** |  |  |  |  |  |  |  |
| --- | --- | --- | --- | --- | --- | --- | --- | --- |
| **Population** | **AC1.195** | **AC1.197** | **AC1.199** | **AC1.201** | **AC1.203** | **AC1.207** | **AC1.209** | **AC1.211** |
| Madera, California, USA | 0.0000 | 0.4935 | 0.0130 | 0.0844 | 0.0000 | 0.0000 | 0.4091 | 0.0000 |
| Fresno, California, USA | 0.2053 | 0.4053 | 0.0000 | 0.2368 | 0.0000 | 0.0000 | 0.1421 | 0.0105 |
| San Mateo County, California, USA | 0.0000 | 0.8125 | 0.0000 | 0.0313 | 0.0000 | 0.0000 | 0.1563 | 0.0000 |
| Amacuzac, Morelos, MEX | 0.0000 | 0.6111 | 0.0278 | 0.1574 | 0.0000 | 0.0000 | 0.2037 | 0.0000 |
| Tucson (TJC2), Arizona, USA | 0.0370 | 0.7037 | 0.0000 | 0.1204 | 0.0000 | 0.0000 | 0.1389 | 0.0000 |
| Mazatan, Chiapas, MEX | 0.0556 | 0.3889 | 0.0000 | 0.0444 | 0.0000 | 0.0000 | 0.5111 | 0.0000 |
| Muscogee County, Georgia, USA | 0.1818 | 0.2455 | 0.0273 | 0.1727 | 0.1455 | 0.0000 | 0.2273 | 0.0000 |
| Tucson, Arizona, USA | 0.0000 | 0.6111 | 0.0000 | 0.0833 | 0.0000 | 0.0000 | 0.3056 | 0.0000 |
| Tapachula, Chiapas, MEX | 0.0556 | 0.4352 | 0.0000 | 0.0370 | 0.0000 | 0.0000 | 0.4722 | 0.0000 |
| Maricopa County, Arizona, USA | 0.0283 | 0.4717 | 0.0000 | 0.1321 | 0.0000 | 0.0000 | 0.3679 | 0.0000 |
| Hermosillo, Sonora, MEX | 0.1100 | 0.3800 | 0.0000 | 0.2600 | 0.0300 | 0.0000 | 0.2200 | 0.0000 |
| Nogales, Sonora, MEX | 0.0980 | 0.3627 | 0.0000 | 0.1961 | 0.0000 | 0.0000 | 0.3431 | 0.0000 |
| Tijuana, Baja California Norte, MEX | 0.0000 | 0.2250 | 0.0000 | 0.3250 | 0.0000 | 0.0000 | 0.4500 | 0.0000 |
| Houston 2009, Texas, USA | 0.3621 | 0.0862 | 0.0000 | 0.2414 | 0.0000 | 0.0000 | 0.3103 | 0.0000 |
| Houston 2011, Texas, USA | 0.0000 | 0.3684 | 0.0000 | 0.0000 | 0.0000 | 0.0000 | 0.6053 | 0.0263 |
| New Orleans, New Orleans, USA | 0.3478 | 0.4457 | 0.0000 | 0.0978 | 0.0000 | 0.0109 | 0.0978 | 0.0000 |
| Miami, Florida, USA | 0.2447 | 0.3511 | 0.0000 | 0.0213 | 0.0000 | 0.0000 | 0.3723 | 0.0106 |
| Vaca Key, Florida, USA | 0.2500 | 0.0952 | 0.0000 | 0.0000 | 0.0000 | 0.0000 | 0.6548 | 0.0000 |
| Pijijiapan, Chiapas, MEX | 0.0851 | 0.3936 | 0.0000 | 0.0000 | 0.0000 | 0.0000 | 0.5213 | 0.0000 |
| Coatzacoalcos, Veracruz, MEX | 0.3200 | 0.0000 | 0.0000 | 0.1200 | 0.0000 | 0.0000 | 0.5600 | 0.0000 |
| Puerto Rico | 0.0926 | 0.1481 | 0.0093 | 0.1204 | 0.0000 | 0.0278 | 0.6019 | 0.0000 |
| Cali, COL | 0.0313 | 0.1625 | 0.0000 | 0.2000 | 0.0000 | 0.0000 | 0.6063 | 0.0000 |
| Cachoeiro, BRA | 0.0286 | 0.2500 | 0.0000 | 0.1071 | 0.0000 | 0.0143 | 0.6000 | 0.0000 |
| Maraba, BRA | 0.0104 | 0.2813 | 0.1146 | 0.0313 | 0.0000 | 0.0000 | 0.5625 | 0.0000 |
| Natal, BRA | 0.0106 | 0.3298 | 0.0000 | 0.2234 | 0.0000 | 0.0000 | 0.4255 | 0.0106 |
| Jacobina, BRA | 0.0106 | 0.1968 | 0.0000 | 0.1383 | 0.0000 | 0.0000 | 0.6543 | 0.0000 |
| Bolivar, VEN | 0.0208 | 0.2917 | 0.0000 | 0.1875 | 0.0000 | 0.0313 | 0.4688 | 0.0000 |
| Dominica, DOM | 0.0000 | 0.1789 | 0.0000 | 0.3316 | 0.0000 | 0.0000 | 0.4895 | 0.0000 |
| Jeddah, SA | 0.1905 | 0.5000 | 0.0298 | 0.0000 | 0.0000 | 0.0774 | 0.2024 | 0.0000 |
| Prachuabkhirikan, THA | 0.1064 | 0.2447 | 0.2021 | 0.0000 | 0.0106 | 0.0000 | 0.4362 | 0.0000 |
| Bangkok, THA | 0.0000 | 0.1531 | 0.0000 | 0.0000 | 0.0000 | 0.0000 | 0.8469 | 0.0000 |
| Cairns, AU | 0.6771 | 0.2917 | 0.0208 | 0.0000 | 0.0000 | 0.0000 | 0.0104 | 0.0000 |
| Tahiti, FP | 0.4375 | 0.2604 | 0.0000 | 0.0000 | 0.1250 | 0.0000 | 0.1771 | 0.0000 |

|  | **AC2 locus** |  |  |  |  |  |
| --- | --- | --- | --- | --- | --- | --- |
| **Population** | **AC2.182** | **AC2.184** | **AC2.186** | **AC2.188** | **AC2.190** | **AC2.192** |
| Madera, California, USA | 0.0000 | 0.0921 | 0.0000 | 0.8684 | 0.0395 | 0.0000 |
| Fresno, California, USA | 0.0000 | 0.1576 | 0.1793 | 0.6630 | 0.0000 | 0.0000 |
| San Mateo County, California, USA | 0.0000 | 0.0625 | 0.0000 | 0.8750 | 0.0625 | 0.0000 |
| Amacuzac, Morelos, MEX | 0.0000 | 0.0556 | 0.2963 | 0.6481 | 0.0000 | 0.0000 |
| Tucson (TJC2), Arizona, USA | 0.0000 | 0.2500 | 0.1667 | 0.5833 | 0.0000 | 0.0000 |
| Mazatan, Chiapas, MEX | 0.0000 | 0.1444 | 0.4222 | 0.4333 | 0.0000 | 0.0000 |
| Muscogee County, Georgia, USA | 0.0000 | 0.3727 | 0.2818 | 0.3455 | 0.0000 | 0.0000 |
| Tucson, Arizona, USA | 0.0000 | 0.4630 | 0.0463 | 0.4907 | 0.0000 | 0.0000 |
| Tapachula, Chiapas, MEX | 0.0000 | 0.1667 | 0.3519 | 0.4815 | 0.0000 | 0.0000 |
| Maricopa County, Arizona, USA | 0.0000 | 0.2736 | 0.0755 | 0.6509 | 0.0000 | 0.0000 |
| Hermosillo, Sonora, MEX | 0.0000 | 0.2400 | 0.0400 | 0.7200 | 0.0000 | 0.0000 |
| Nogales, Sonora, MEX | 0.0000 | 0.6176 | 0.1176 | 0.2647 | 0.0000 | 0.0000 |
| Tijuana, Baja California Norte, MEX | 0.0000 | 0.4500 | 0.3000 | 0.2500 | 0.0000 | 0.0000 |
| Houston 2009, Texas, USA | 0.0000 | 0.0172 | 0.0517 | 0.9310 | 0.0000 | 0.0000 |
| Houston 2011, Texas, USA | 0.0000 | 0.2368 | 0.0263 | 0.7368 | 0.0000 | 0.0000 |
| New Orleans, New Orleans, USA | 0.0000 | 0.1667 | 0.1190 | 0.7143 | 0.0000 | 0.0000 |
| Miami, Florida, USA | 0.0213 | 0.2021 | 0.0957 | 0.6809 | 0.0000 | 0.0000 |
| Vaca Key, Florida, USA | 0.0000 | 0.2619 | 0.0119 | 0.7262 | 0.0000 | 0.0000 |
| Pijijiapan, Chiapas, MEX | 0.0000 | 0.0426 | 0.4894 | 0.4681 | 0.0000 | 0.0000 |
| Coatzacoalcos, Veracruz, MEX | 0.0000 | 0.0100 | 0.2100 | 0.7800 | 0.0000 | 0.0000 |
| Puerto Rico | 0.0000 | 0.6759 | 0.1667 | 0.1574 | 0.0000 | 0.0000 |
| Cali, COL | 0.0000 | 0.3125 | 0.0000 | 0.6875 | 0.0000 | 0.0000 |
| Cachoeiro, BRA | 0.0000 | 0.1929 | 0.1714 | 0.6357 | 0.0000 | 0.0000 |
| Maraba, BRA | 0.0000 | 0.2292 | 0.0521 | 0.7188 | 0.0000 | 0.0000 |
| Natal, BRA | 0.0000 | 0.4894 | 0.0319 | 0.4787 | 0.0000 | 0.0000 |
| Jacobina, BRA | 0.0000 | 0.1223 | 0.1436 | 0.7287 | 0.0053 | 0.0000 |
| Bolivar, VEN | 0.0000 | 0.1354 | 0.0833 | 0.7813 | 0.0000 | 0.0000 |
| Dominica, DOM | 0.0000 | 0.0053 | 0.0000 | 0.8211 | 0.1737 | 0.0000 |
| Jeddah, SA | 0.0000 | 0.1786 | 0.0417 | 0.6548 | 0.0774 | 0.0476 |
| Prachuabkhirikan, THA | 0.0000 | 0.1809 | 0.0851 | 0.7340 | 0.0000 | 0.0000 |
| Bangkok, THA | 0.0000 | 0.1100 | 0.0000 | 0.8900 | 0.0000 | 0.0000 |
| Cairns, AU | 0.0000 | 0.6979 | 0.0000 | 0.1771 | 0.1250 | 0.0000 |
| Tahiti, FP | 0.0000 | 0.5625 | 0.1354 | 0.0833 | 0.2188 | 0.0000 |

|  | **AC4 locus** |  |  |  |
| --- | --- | --- | --- | --- |
| **Population** | **AC4.128** | **AC4.130** | **AC4.132** | **AC4.134** |
| Madera, California, USA | 0.5649 | 0.4351 | 0.0000 | 0.0000 |
| Fresno, California, USA | 0.9526 | 0.0474 | 0.0000 | 0.0000 |
| San Mateo County, California, USA | 0.4000 | 0.6000 | 0.0000 | 0.0000 |
| Amacuzac, Morelos, MEX | 0.6667 | 0.3148 | 0.0185 | 0.0000 |
| Tucson (TJC2), Arizona, USA | 0.9352 | 0.0648 | 0.0000 | 0.0000 |
| Mazatan, Chiapas, MEX | 0.8667 | 0.1333 | 0.0000 | 0.0000 |
| Muscogee County, Georgia, USA | 0.5370 | 0.4630 | 0.0000 | 0.0000 |
| Tucson, Arizona, USA | 0.8241 | 0.1759 | 0.0000 | 0.0000 |
| Tapachula, Chiapas, MEX | 0.7500 | 0.2500 | 0.0000 | 0.0000 |
| Maricopa County, Arizona, USA | 0.8774 | 0.1226 | 0.0000 | 0.0000 |
| Hermosillo, Sonora, MEX | 0.7700 | 0.2300 | 0.0000 | 0.0000 |
| Nogales, Sonora, MEX | 0.7941 | 0.2059 | 0.0000 | 0.0000 |
| Tijuana, Baja California Norte, MEX | 0.6250 | 0.3750 | 0.0000 | 0.0000 |
| Houston 2009, Texas, USA | 0.7069 | 0.2759 | 0.0000 | 0.0172 |
| Houston 2011, Texas, USA | 0.8421 | 0.1579 | 0.0000 | 0.0000 |
| New Orleans, New Orleans, USA | 0.5870 | 0.4130 | 0.0000 | 0.0000 |
| Miami, Florida, USA | 0.4787 | 0.5213 | 0.0000 | 0.0000 |
| Vaca Key, Florida, USA | 0.4643 | 0.5357 | 0.0000 | 0.0000 |
| Pijijiapan, Chiapas, MEX | 0.9255 | 0.0745 | 0.0000 | 0.0000 |
| Coatzacoalcos, Veracruz, MEX | 0.9100 | 0.0900 | 0.0000 | 0.0000 |
| Puerto Rico | 0.7222 | 0.2778 | 0.0000 | 0.0000 |
| Cali, COL | 0.5375 | 0.4625 | 0.0000 | 0.0000 |
| Cachoeiro, BRA | 0.8714 | 0.1286 | 0.0000 | 0.0000 |
| Maraba, BRA | 0.5833 | 0.4167 | 0.0000 | 0.0000 |
| Natal, BRA | 0.5532 | 0.4468 | 0.0000 | 0.0000 |
| Jacobina, BRA | 0.7606 | 0.2394 | 0.0000 | 0.0000 |
| Bolivar, VEN | 0.8958 | 0.1042 | 0.0000 | 0.0000 |
| Dominica, DOM | 0.9579 | 0.0421 | 0.0000 | 0.0000 |
| Jeddah, SA | 0.6369 | 0.3631 | 0.0000 | 0.0000 |
| Prachuabkhirikan, THA | 0.6277 | 0.3723 | 0.0000 | 0.0000 |
| Bangkok, THA | 0.5000 | 0.5000 | 0.0000 | 0.0000 |
| Cairns, AU | 0.1875 | 0.8125 | 0.0000 | 0.0000 |
| Tahiti, FP | 0.7083 | 0.2917 | 0.0000 | 0.0000 |

|  | **AC5 locus** |  |  |  |  |  |  |  |  |  |
| --- | --- | --- | --- | --- | --- | --- | --- | --- | --- | --- |
| **Population** | **AC5.145** | **AC5.147** | **AC5.148** | **AC5.149** | **AC5.151** | **AC5.152** | **AC5.153** | **AC5.154** | **AC5.155** | **AC5.156** |
| Madera, California, USA | 0.0000 | 0.0000 | 0.2078 | 0.0000 | 0.0000 | 0.0000 | 0.0000 | 0.0260 | 0.0000 | 0.1104 |
| Fresno, California, USA | 0.0000 | 0.0000 | 0.0316 | 0.0000 | 0.0000 | 0.0000 | 0.0000 | 0.0368 | 0.0000 | 0.1632 |
| San Mateo County, California, USA | 0.0000 | 0.0000 | 0.1563 | 0.0000 | 0.0000 | 0.0000 | 0.0000 | 0.0000 | 0.0000 | 0.1563 |
| Amacuzac, Morelos, MEX | 0.0000 | 0.0000 | 0.0000 | 0.0000 | 0.0000 | 0.1389 | 0.0000 | 0.0000 | 0.0000 | 0.4074 |
| Tucson (TJC2), Arizona, USA | 0.0000 | 0.0000 | 0.2593 | 0.0000 | 0.0000 | 0.0000 | 0.0000 | 0.0000 | 0.0000 | 0.0278 |
| Mazatan, Chiapas, MEX | 0.0000 | 0.0000 | 0.0000 | 0.0000 | 0.0000 | 0.0000 | 0.0000 | 0.0000 | 0.0000 | 0.0000 |
| Muscogee County, Georgia, USA | 0.0182 | 0.1091 | 0.0000 | 0.0273 | 0.0000 | 0.0000 | 0.0000 | 0.0182 | 0.0000 | 0.0636 |
| Tucson, Arizona, USA | 0.0000 | 0.0093 | 0.0278 | 0.0000 | 0.0000 | 0.0741 | 0.0000 | 0.0000 | 0.0000 | 0.0926 |
| Tapachula, Chiapas, MEX | 0.0000 | 0.0000 | 0.0000 | 0.0000 | 0.0000 | 0.0000 | 0.0000 | 0.0000 | 0.0000 | 0.0000 |
| Maricopa County, Arizona, USA | 0.0000 | 0.0094 | 0.0755 | 0.0000 | 0.0000 | 0.0000 | 0.0000 | 0.0000 | 0.0000 | 0.0849 |
| Hermosillo, Sonora, MEX | 0.0000 | 0.0000 | 0.0000 | 0.0000 | 0.0000 | 0.2400 | 0.0000 | 0.0000 | 0.0100 | 0.1300 |
| Nogales, Sonora, MEX | 0.0000 | 0.0000 | 0.0000 | 0.0000 | 0.0000 | 0.0588 | 0.0000 | 0.0000 | 0.0000 | 0.0882 |
| Tijuana, Baja California Norte, MEX | 0.0000 | 0.0000 | 0.0000 | 0.0000 | 0.0000 | 0.0000 | 0.0000 | 0.0000 | 0.0000 | 0.1500 |
| Houston 2009, Texas, USA | 0.0000 | 0.1207 | 0.0000 | 0.0000 | 0.0000 | 0.0172 | 0.0000 | 0.0000 | 0.0000 | 0.0690 |
| Houston 2011, Texas, USA | 0.0000 | 0.1053 | 0.0000 | 0.0000 | 0.0000 | 0.0000 | 0.0000 | 0.0000 | 0.0000 | 0.0526 |
| New Orleans, New Orleans, USA | 0.0000 | 0.2000 | 0.0333 | 0.0000 | 0.0000 | 0.0000 | 0.0000 | 0.0222 | 0.0111 | 0.1556 |
| Miami, Florida, USA | 0.0000 | 0.1915 | 0.0106 | 0.0000 | 0.0000 | 0.0532 | 0.0000 | 0.1170 | 0.0000 | 0.2660 |
| Vaca Key, Florida, USA | 0.0000 | 0.1429 | 0.0952 | 0.0000 | 0.0000 | 0.0119 | 0.0000 | 0.0357 | 0.0000 | 0.1905 |
| Pijijiapan, Chiapas, MEX | 0.0000 | 0.0000 | 0.0000 | 0.0000 | 0.0000 | 0.0000 | 0.0000 | 0.0000 | 0.0000 | 0.0532 |
| Coatzacoalcos, Veracruz, MEX | 0.0000 | 0.0000 | 0.0000 | 0.0000 | 0.0000 | 0.0000 | 0.0000 | 0.0000 | 0.0000 | 0.0000 |
| Puerto Rico | 0.0000 | 0.1019 | 0.0185 | 0.0000 | 0.0000 | 0.0000 | 0.0000 | 0.0000 | 0.0000 | 0.3241 |
| Cali, COL | 0.0000 | 0.0375 | 0.0000 | 0.0000 | 0.0000 | 0.4625 | 0.0000 | 0.0000 | 0.0000 | 0.0688 |
| Cachoeiro, BRA | 0.0000 | 0.0214 | 0.0000 | 0.0000 | 0.0071 | 0.0000 | 0.0000 | 0.0000 | 0.0000 | 0.1857 |
| Maraba, BRA | 0.0000 | 0.0000 | 0.0000 | 0.0000 | 0.0000 | 0.0417 | 0.0000 | 0.0000 | 0.0000 | 0.0000 |
| Natal, BRA | 0.0000 | 0.0638 | 0.0000 | 0.0000 | 0.0000 | 0.0000 | 0.0000 | 0.0000 | 0.0000 | 0.2340 |
| Jacobina, BRA | 0.0000 | 0.0000 | 0.0000 | 0.0000 | 0.0000 | 0.0000 | 0.0000 | 0.0000 | 0.0000 | 0.1398 |
| Bolivar, VEN | 0.0000 | 0.0625 | 0.0000 | 0.0000 | 0.0000 | 0.0000 | 0.0000 | 0.0000 | 0.0313 | 0.0417 |
| Dominica, DOM | 0.0000 | 0.0053 | 0.0000 | 0.0000 | 0.0000 | 0.0000 | 0.0000 | 0.0000 | 0.2632 | 0.3947 |
| Jeddah, SA | 0.0000 | 0.0833 | 0.0000 | 0.0000 | 0.0000 | 0.0000 | 0.0000 | 0.0298 | 0.1369 | 0.3036 |
| Prachuabkhirikan, THA | 0.0000 | 0.0000 | 0.0957 | 0.0000 | 0.0000 | 0.1170 | 0.0000 | 0.0426 | 0.0000 | 0.1596 |
| Bangkok, THA | 0.0000 | 0.0000 | 0.1837 | 0.0000 | 0.0000 | 0.0000 | 0.0000 | 0.0000 | 0.0000 | 0.1633 |
| Cairns, AU | 0.0000 | 0.0000 | 0.0000 | 0.0000 | 0.0000 | 0.0000 | 0.0104 | 0.0000 | 0.0000 | 0.2396 |
| Tahiti, FP | 0.0000 | 0.0417 | 0.0000 | 0.0000 | 0.0000 | 0.0000 | 0.0000 | 0.0000 | 0.0000 | 0.6458 |

|  |  | **AC5 locus** | |  |  |  |  |  |  |  |  |  |
| --- | --- | --- | --- | --- | --- | --- | --- | --- | --- | --- | --- | --- |
| **Population** |  | **AC5.157** | **AC5.158** | | **AC5.159** | **AC5.160** | **AC5.161** | **AC5.162** | **AC5.163** | **AC5.164** | **AC5.165** | **AC5.169** |
| Madera, California, USA |  | 0.0260 | 0.2338 | | 0.2662 | 0.1169 | 0.0130 | 0.0000 | 0.0000 | 0.0000 | 0.0000 | 0.0000 |
| Fresno, California, USA |  | 0.0842 | 0.4000 | | 0.0000 | 0.0737 | 0.0421 | 0.0000 | 0.0000 | 0.1684 | 0.0000 | 0.0000 |
| San Mateo County, California, USA |  | 0.1250 | 0.2188 | | 0.3438 | 0.0000 | 0.0000 | 0.0000 | 0.0000 | 0.0000 | 0.0000 | 0.0000 |
| Amacuzac, Morelos, MEX |  | 0.0093 | 0.0463 | | 0.1204 | 0.2685 | 0.0093 | 0.0000 | 0.0000 | 0.0000 | 0.0000 | 0.0000 |
| Tucson (TJC2), Arizona, USA |  | 0.0185 | 0.3333 | | 0.2778 | 0.0000 | 0.0833 | 0.0000 | 0.0000 | 0.0000 | 0.0000 | 0.0000 |
| Mazatan, Chiapas, MEX |  | 0.0000 | 0.2889 | | 0.0000 | 0.0000 | 0.6556 | 0.0000 | 0.0556 | 0.0000 | 0.0000 | 0.0000 |
| Muscogee County, Georgia, USA |  | 0.2364 | 0.1364 | | 0.2182 | 0.0273 | 0.1364 | 0.0091 | 0.0000 | 0.0000 | 0.0000 | 0.0000 |
| Tucson, Arizona, USA |  | 0.2500 | 0.2222 | | 0.2500 | 0.0000 | 0.0741 | 0.0000 | 0.0000 | 0.0000 | 0.0000 | 0.0000 |
| Tapachula, Chiapas, MEX |  | 0.0000 | 0.3796 | | 0.1296 | 0.0000 | 0.4537 | 0.0000 | 0.0278 | 0.0093 | 0.0000 | 0.0000 |
| Maricopa County, Arizona, USA |  | 0.0849 | 0.1604 | | 0.4340 | 0.0377 | 0.1132 | 0.0000 | 0.0000 | 0.0000 | 0.0000 | 0.0000 |
| Hermosillo, Sonora, MEX |  | 0.0400 | 0.3600 | | 0.1800 | 0.0000 | 0.0400 | 0.0000 | 0.0000 | 0.0000 | 0.0000 | 0.0000 |
| Nogales, Sonora, MEX |  | 0.0392 | 0.4412 | | 0.1667 | 0.0000 | 0.1961 | 0.0000 | 0.0098 | 0.0000 | 0.0000 | 0.0000 |
| Tijuana, Baja California Norte, MEX |  | 0.2500 | 0.3000 | | 0.0000 | 0.3000 | 0.0000 | 0.0000 | 0.0000 | 0.0000 | 0.0000 | 0.0000 |
| Houston 2009, Texas, USA |  | 0.2931 | 0.0345 | | 0.0172 | 0.4483 | 0.0000 | 0.0000 | 0.0000 | 0.0000 | 0.0000 | 0.0000 |
| Houston 2011, Texas, USA |  | 0.2368 | 0.2632 | | 0.3421 | 0.0000 | 0.0000 | 0.0000 | 0.0000 | 0.0000 | 0.0000 | 0.0000 |
| New Orleans, New Orleans, USA |  | 0.0667 | 0.1333 | | 0.1778 | 0.0667 | 0.0444 | 0.0111 | 0.0333 | 0.0444 | 0.0000 | 0.0000 |
| Miami, Florida, USA |  | 0.0319 | 0.0957 | | 0.0957 | 0.0532 | 0.0213 | 0.0319 | 0.0000 | 0.0106 | 0.0213 | 0.0000 |
| Vaca Key, Florida, USA |  | 0.1071 | 0.1071 | | 0.1429 | 0.0595 | 0.0833 | 0.0000 | 0.0000 | 0.0000 | 0.0000 | 0.0238 |
| Pijijiapan, Chiapas, MEX |  | 0.0000 | 0.1702 | | 0.1277 | 0.0000 | 0.5957 | 0.0000 | 0.0532 | 0.0000 | 0.0000 | 0.0000 |
| Coatzacoalcos, Veracruz, MEX |  | 0.2500 | 0.0000 | | 0.3500 | 0.1100 | 0.2900 | 0.0000 | 0.0000 | 0.0000 | 0.0000 | 0.0000 |
| Puerto Rico |  | 0.0556 | 0.1111 | | 0.3241 | 0.0648 | 0.0000 | 0.0000 | 0.0000 | 0.0000 | 0.0000 | 0.0000 |
| Cali, COL |  | 0.0000 | 0.2125 | | 0.1313 | 0.0875 | 0.0000 | 0.0000 | 0.0000 | 0.0000 | 0.0000 | 0.0000 |
| Cachoeiro, BRA |  | 0.0643 | 0.0143 | | 0.6714 | 0.0357 | 0.0000 | 0.0000 | 0.0000 | 0.0000 | 0.0000 | 0.0000 |
| Maraba, BRA |  | 0.0000 | 0.5521 | | 0.1250 | 0.2813 | 0.0000 | 0.0000 | 0.0000 | 0.0000 | 0.0000 | 0.0000 |
| Natal, BRA |  | 0.0213 | 0.0000 | | 0.6809 | 0.0000 | 0.0000 | 0.0000 | 0.0000 | 0.0000 | 0.0000 | 0.0000 |
| Jacobina, BRA |  | 0.0000 | 0.0269 | | 0.8280 | 0.0000 | 0.0054 | 0.0000 | 0.0000 | 0.0000 | 0.0000 | 0.0000 |
| Bolivar, VEN |  | 0.0313 | 0.2292 | | 0.4167 | 0.1563 | 0.0104 | 0.0208 | 0.0000 | 0.0000 | 0.0000 | 0.0000 |
| Dominica, DOM |  | 0.0368 | 0.0368 | | 0.2632 | 0.0000 | 0.0000 | 0.0000 | 0.0000 | 0.0000 | 0.0000 | 0.0000 |
| Jeddah, SA |  | 0.0000 | 0.1310 | | 0.2321 | 0.0060 | 0.0655 | 0.0060 | 0.0060 | 0.0000 | 0.0000 | 0.0000 |
| Prachuabkhirikan, THA |  | 0.0000 | 0.0532 | | 0.4362 | 0.0000 | 0.0319 | 0.0000 | 0.0000 | 0.0000 | 0.0638 | 0.0000 |
| Bangkok, THA |  | 0.2245 | 0.3980 | | 0.0306 | 0.0000 | 0.0000 | 0.0000 | 0.0000 | 0.0000 | 0.0000 | 0.0000 |
| Cairns, AU |  | 0.4375 | 0.0000 | | 0.1563 | 0.0208 | 0.0000 | 0.0000 | 0.1354 | 0.0000 | 0.0000 | 0.0000 |
| Tahiti, FP |  | 0.0729 | 0.0729 | | 0.1250 | 0.0104 | 0.0313 | 0.0000 | 0.0000 | 0.0000 | 0.0000 | 0.0000 |

|  | **CT2 locus** |  |  |  |  |  |  |
| --- | --- | --- | --- | --- | --- | --- | --- |
| **Population** | **CT2.182** | **CT2.184** | **CT2.186** | **CT2.188** | **CT2.190** | **CT2.194** | **CT2.196** |
| Madera, California, USA | 0.0000 | 0.7500 | 0.0000 | 0.2500 | 0.0000 | 0.0000 | 0.0000 |
| Fresno, California, USA | 0.0000 | 0.9211 | 0.0000 | 0.0789 | 0.0000 | 0.0000 | 0.0000 |
| San Mateo County, California, USA | 0.0000 | 0.9063 | 0.0000 | 0.0938 | 0.0000 | 0.0000 | 0.0000 |
| Amacuzac, Morelos, MEX | 0.0000 | 0.9907 | 0.0000 | 0.0093 | 0.0000 | 0.0000 | 0.0000 |
| Tucson (TJC2), Arizona, USA | 0.0000 | 0.5093 | 0.0000 | 0.4907 | 0.0000 | 0.0000 | 0.0000 |
| Mazatan, Chiapas, MEX | 0.0000 | 1.0000 | 0.0000 | 0.0000 | 0.0000 | 0.0000 | 0.0000 |
| Muscogee County, Georgia, USA | 0.0000 | 0.4545 | 0.0000 | 0.5455 | 0.0000 | 0.0000 | 0.0000 |
| Tucson, Arizona, USA | 0.0000 | 0.6759 | 0.0000 | 0.3241 | 0.0000 | 0.0000 | 0.0000 |
| Tapachula, Chiapas, MEX | 0.0000 | 1.0000 | 0.0000 | 0.0000 | 0.0000 | 0.0000 | 0.0000 |
| Maricopa County, Arizona, USA | 0.0000 | 0.5660 | 0.0000 | 0.4340 | 0.0000 | 0.0000 | 0.0000 |
| Hermosillo, Sonora, MEX | 0.0000 | 0.7200 | 0.0000 | 0.2800 | 0.0000 | 0.0000 | 0.0000 |
| Nogales, Sonora, MEX | 0.0000 | 0.4804 | 0.0000 | 0.5196 | 0.0000 | 0.0000 | 0.0000 |
| Tijuana, Baja California Norte, MEX | 0.0000 | 0.6000 | 0.0000 | 0.4000 | 0.0000 | 0.0000 | 0.0000 |
| Houston 2009, Texas, USA | 0.0000 | 0.7069 | 0.0000 | 0.2931 | 0.0000 | 0.0000 | 0.0000 |
| Houston 2011, Texas, USA | 0.0000 | 1.0000 | 0.0000 | 0.0000 | 0.0000 | 0.0000 | 0.0000 |
| New Orleans, New Orleans, USA | 0.0000 | 0.7000 | 0.0000 | 0.3000 | 0.0000 | 0.0000 | 0.0000 |
| Miami, Florida, USA | 0.0000 | 0.6413 | 0.0000 | 0.3478 | 0.0000 | 0.0109 | 0.0000 |
| Vaca Key, Florida, USA | 0.0000 | 0.7738 | 0.0000 | 0.2262 | 0.0000 | 0.0000 | 0.0000 |
| Pijijiapan, Chiapas, MEX | 0.0000 | 1.0000 | 0.0000 | 0.0000 | 0.0000 | 0.0000 | 0.0000 |
| Coatzacoalcos, Veracruz, MEX | 0.0000 | 1.0000 | 0.0000 | 0.0000 | 0.0000 | 0.0000 | 0.0000 |
| Puerto Rico | 0.0000 | 0.6887 | 0.0000 | 0.3113 | 0.0000 | 0.0000 | 0.0000 |
| Cali, COL | 0.0063 | 0.2688 | 0.0125 | 0.7125 | 0.0000 | 0.0000 | 0.0000 |
| Cachoeiro, BRA | 0.0000 | 0.6929 | 0.0000 | 0.3000 | 0.0000 | 0.0071 | 0.0000 |
| Maraba, BRA | 0.0000 | 0.4271 | 0.0000 | 0.4688 | 0.0000 | 0.1042 | 0.0000 |
| Natal, BRA | 0.0000 | 0.9468 | 0.0000 | 0.0532 | 0.0000 | 0.0000 | 0.0000 |
| Jacobina, BRA | 0.0000 | 0.9043 | 0.0000 | 0.0957 | 0.0000 | 0.0000 | 0.0000 |
| Bolivar, VEN | 0.0000 | 0.3854 | 0.0000 | 0.6146 | 0.0000 | 0.0000 | 0.0000 |
| Dominica, DOM | 0.0000 | 0.9947 | 0.0000 | 0.0053 | 0.0000 | 0.0000 | 0.0000 |
| Jeddah, SA | 0.0000 | 0.3869 | 0.0000 | 0.5179 | 0.0000 | 0.0655 | 0.0298 |
| Prachuabkhirikan, THA | 0.0000 | 0.1596 | 0.0000 | 0.6170 | 0.2128 | 0.0106 | 0.0000 |
| Bangkok, THA | 0.0000 | 0.3400 | 0.0000 | 0.6600 | 0.0000 | 0.0000 | 0.0000 |
| Cairns, AU | 0.0000 | 0.6146 | 0.0000 | 0.3854 | 0.0000 | 0.0000 | 0.0000 |
| Tahiti, FP | 0.0000 | 0.3229 | 0.0000 | 0.6042 | 0.0000 | 0.0729 | 0.0000 |

|  | **AG1 locus** |  |  |  |  |  |  |
| --- | --- | --- | --- | --- | --- | --- | --- |
| **Population** | **AG1.113** | **AG1.115** | **AG1.117** | **AG1.119** | **AG1.121** | **AG1.123** | **AG1.127** |
| Madera, California, USA | 0.0000 | 0.0909 | 0.1558 | 0.0000 | 0.4221 | 0.0000 | 0.3312 |
| Fresno, California, USA | 0.0000 | 0.2316 | 0.4684 | 0.2105 | 0.0000 | 0.0000 | 0.0895 |
| San Mateo County, California, USA | 0.0000 | 0.0625 | 0.2500 | 0.0000 | 0.3125 | 0.0000 | 0.3750 |
| Amacuzac, Morelos, MEX | 0.0000 | 0.2963 | 0.2593 | 0.2037 | 0.2407 | 0.0000 | 0.0000 |
| Tucson (TJC2), Arizona, USA | 0.0000 | 0.6296 | 0.0926 | 0.2685 | 0.0093 | 0.0000 | 0.0000 |
| Mazatan, Chiapas, MEX | 0.0000 | 0.2222 | 0.3333 | 0.0889 | 0.3556 | 0.0000 | 0.0000 |
| Muscogee County, Georgia, USA | 0.0000 | 0.5093 | 0.2500 | 0.1204 | 0.1204 | 0.0000 | 0.0000 |
| Tucson, Arizona, USA | 0.0000 | 0.6296 | 0.1389 | 0.1389 | 0.0926 | 0.0000 | 0.0000 |
| Tapachula, Chiapas, MEX | 0.0000 | 0.2037 | 0.1944 | 0.0833 | 0.5185 | 0.0000 | 0.0000 |
| Maricopa County, Arizona, USA | 0.0000 | 0.4245 | 0.3113 | 0.1887 | 0.0755 | 0.0000 | 0.0000 |
| Hermosillo, Sonora, MEX | 0.0000 | 0.4300 | 0.1900 | 0.1800 | 0.2000 | 0.0000 | 0.0000 |
| Nogales, Sonora, MEX | 0.0000 | 0.6275 | 0.1569 | 0.0980 | 0.1176 | 0.0000 | 0.0000 |
| Tijuana, Baja California Norte, MEX | 0.0000 | 0.1250 | 0.6750 | 0.2000 | 0.0000 | 0.0000 | 0.0000 |
| Houston 2009, Texas, USA | 0.0000 | 0.0172 | 0.0000 | 0.1897 | 0.7931 | 0.0000 | 0.0000 |
| Houston 2011, Texas, USA | 0.0000 | 0.2632 | 0.3947 | 0.1053 | 0.1842 | 0.0000 | 0.0526 |
| New Orleans, New Orleans, USA | 0.0000 | 0.2283 | 0.3152 | 0.1630 | 0.2935 | 0.0000 | 0.0000 |
| Miami, Florida, USA | 0.0000 | 0.2234 | 0.2872 | 0.2234 | 0.2553 | 0.0106 | 0.0000 |
| Vaca Key, Florida, USA | 0.0000 | 0.1905 | 0.3214 | 0.1429 | 0.3452 | 0.0000 | 0.0000 |
| Pijijiapan, Chiapas, MEX | 0.0000 | 0.3261 | 0.1739 | 0.0870 | 0.4130 | 0.0000 | 0.0000 |
| Coatzacoalcos, Veracruz, MEX | 0.0000 | 0.2500 | 0.0600 | 0.6900 | 0.0000 | 0.0000 | 0.0000 |
| Puerto Rico | 0.0000 | 0.1019 | 0.3889 | 0.1574 | 0.3519 | 0.0000 | 0.0000 |
| Cali, COL | 0.0000 | 0.2188 | 0.5813 | 0.0813 | 0.1188 | 0.0000 | 0.0000 |
| Cachoeiro, BRA | 0.0000 | 0.0071 | 0.2714 | 0.4000 | 0.3143 | 0.0071 | 0.0000 |
| Maraba, BRA | 0.0000 | 0.1875 | 0.0521 | 0.3542 | 0.4063 | 0.0000 | 0.0000 |
| Natal, BRA | 0.0000 | 0.1596 | 0.3617 | 0.1383 | 0.3404 | 0.0000 | 0.0000 |
| Jacobina, BRA | 0.0269 | 0.0000 | 0.6237 | 0.0753 | 0.2742 | 0.0000 | 0.0000 |
| Bolivar, VEN | 0.0000 | 0.2083 | 0.3750 | 0.1458 | 0.2708 | 0.0000 | 0.0000 |
| Dominica, DOM | 0.0000 | 0.0579 | 0.5842 | 0.1368 | 0.2211 | 0.0000 | 0.0000 |
| Jeddah, SA | 0.0000 | 0.3690 | 0.2262 | 0.1667 | 0.2381 | 0.0000 | 0.0000 |
| Prachuabkhirikan, THA | 0.0000 | 0.1702 | 0.2234 | 0.1809 | 0.4255 | 0.0000 | 0.0000 |
| Bangkok, THA | 0.0000 | 0.0900 | 0.1200 | 0.0600 | 0.7300 | 0.0000 | 0.0000 |
| Cairns, AU | 0.0000 | 0.0313 | 0.2188 | 0.1771 | 0.3125 | 0.2604 | 0.0000 |
| Tahiti, FP | 0.0000 | 0.4583 | 0.1250 | 0.2396 | 0.1771 | 0.0000 | 0.0000 |

|  | **AG2 locus** |  |  |  |  |  |  |  |  |  |
| --- | --- | --- | --- | --- | --- | --- | --- | --- | --- | --- |
| **Population** | **AG2.113** | **AG2.114** | **AG2.115** | **AG2.117** | **AG2.119** | **AG2.121** | **AG2.127** | **AG2.129** | **AG2.131** | **AG2.133** |
| Madera, California, USA | 0.0000 | 0.0000 | 0.3487 | 0.0000 | 0.0000 | 0.0000 | 0.0000 | 0.0000 | 0.0000 | 0.0658 |
| Fresno, California, USA | 0.0000 | 0.0000 | 0.3387 | 0.0161 | 0.0000 | 0.0000 | 0.0000 | 0.5323 | 0.0000 | 0.0430 |
| San Mateo County, California, USA | 0.0000 | 0.0000 | 0.2500 | 0.0000 | 0.0000 | 0.0000 | 0.0000 | 0.0000 | 0.0000 | 0.1563 |
| Amacuzac, Morelos, MEX | 0.0000 | 0.0000 | 0.1204 | 0.2407 | 0.0000 | 0.2130 | 0.0093 | 0.2500 | 0.0833 | 0.0000 |
| Tucson (TJC2), Arizona, USA | 0.0000 | 0.0000 | 0.4815 | 0.0093 | 0.0000 | 0.0000 | 0.0000 | 0.1481 | 0.2500 | 0.0000 |
| Mazatan, Chiapas, MEX | 0.0000 | 0.0000 | 0.3667 | 0.0000 | 0.1000 | 0.0000 | 0.0000 | 0.0000 | 0.4333 | 0.0889 |
| Muscogee County, Georgia, USA | 0.0000 | 0.0000 | 0.4909 | 0.0818 | 0.0000 | 0.0000 | 0.0000 | 0.0364 | 0.0364 | 0.1818 |
| Tucson, Arizona, USA | 0.0000 | 0.0000 | 0.3241 | 0.0093 | 0.0000 | 0.0000 | 0.0000 | 0.0463 | 0.2500 | 0.1574 |
| Tapachula, Chiapas, MEX | 0.0000 | 0.0000 | 0.2870 | 0.0278 | 0.1019 | 0.0000 | 0.0000 | 0.0370 | 0.4537 | 0.0463 |
| Maricopa County, Arizona, USA | 0.0000 | 0.0000 | 0.6509 | 0.0377 | 0.0000 | 0.0000 | 0.0000 | 0.0189 | 0.1887 | 0.0377 |
| Hermosillo, Sonora, MEX | 0.0000 | 0.0000 | 0.6400 | 0.0100 | 0.0000 | 0.0000 | 0.0000 | 0.0200 | 0.1500 | 0.0000 |
| Nogales, Sonora, MEX | 0.0000 | 0.0000 | 0.5098 | 0.0000 | 0.0000 | 0.0000 | 0.0000 | 0.0000 | 0.2255 | 0.0196 |
| Tijuana, Baja California Norte, MEX | 0.0000 | 0.0000 | 0.0750 | 0.0000 | 0.0000 | 0.0000 | 0.0000 | 0.0000 | 0.0000 | 0.0000 |
| Houston 2009, Texas, USA | 0.0000 | 0.0000 | 0.7414 | 0.2241 | 0.0000 | 0.0000 | 0.0000 | 0.0345 | 0.0000 | 0.0000 |
| Houston 2011, Texas, USA | 0.0000 | 0.0000 | 0.7105 | 0.1053 | 0.0000 | 0.0000 | 0.0000 | 0.1842 | 0.0000 | 0.0000 |
| New Orleans, New Orleans, USA | 0.0000 | 0.0000 | 0.5889 | 0.1000 | 0.0333 | 0.0000 | 0.0000 | 0.1889 | 0.0000 | 0.0111 |
| Miami, Florida, USA | 0.0000 | 0.0233 | 0.3721 | 0.1860 | 0.1279 | 0.0000 | 0.0000 | 0.0581 | 0.0581 | 0.0000 |
| Vaca Key, Florida, USA | 0.0000 | 0.0000 | 0.4048 | 0.1071 | 0.1310 | 0.0000 | 0.0000 | 0.0000 | 0.0000 | 0.0000 |
| Pijijiapan, Chiapas, MEX | 0.0000 | 0.0000 | 0.5851 | 0.0000 | 0.0000 | 0.0000 | 0.0000 | 0.0000 | 0.3511 | 0.0106 |
| Coatzacoalcos, Veracruz, MEX | 0.0000 | 0.0000 | 0.5600 | 0.1200 | 0.0000 | 0.0000 | 0.0000 | 0.0000 | 0.0000 | 0.0000 |
| Puerto Rico | 0.0000 | 0.0000 | 0.3302 | 0.0755 | 0.1509 | 0.0000 | 0.0000 | 0.0000 | 0.0283 | 0.0472 |
| Cali, COL | 0.0000 | 0.0000 | 0.0250 | 0.0500 | 0.6750 | 0.0000 | 0.0000 | 0.0000 | 0.0000 | 0.0000 |
| Cachoeiro, BRA | 0.0000 | 0.0000 | 0.0580 | 0.1087 | 0.4638 | 0.0000 | 0.0000 | 0.0000 | 0.0000 | 0.0000 |
| Maraba, BRA | 0.0000 | 0.0000 | 0.2292 | 0.0208 | 0.4688 | 0.0000 | 0.0000 | 0.0104 | 0.0417 | 0.0000 |
| Natal, BRA | 0.0000 | 0.0000 | 0.0000 | 0.2234 | 0.4149 | 0.0000 | 0.0000 | 0.0000 | 0.0000 | 0.0000 |
| Jacobina, BRA | 0.0000 | 0.0000 | 0.3245 | 0.1489 | 0.2340 | 0.0000 | 0.0000 | 0.0000 | 0.0160 | 0.0000 |
| Bolivar, VEN | 0.0000 | 0.0521 | 0.4583 | 0.1771 | 0.0833 | 0.0000 | 0.0000 | 0.0000 | 0.0000 | 0.0000 |
| Dominica, DOM | 0.0000 | 0.0000 | 0.5789 | 0.0000 | 0.2316 | 0.0000 | 0.0000 | 0.0000 | 0.0000 | 0.0000 |
| Jeddah, SA | 0.0122 | 0.0000 | 0.2134 | 0.3780 | 0.0305 | 0.0183 | 0.0000 | 0.0244 | 0.0000 | 0.0183 |
| Prachuabkhirikan, THA | 0.0000 | 0.0000 | 0.0319 | 0.6383 | 0.1383 | 0.0000 | 0.0000 | 0.0000 | 0.0000 | 0.1064 |
| Bangkok, THA | 0.0000 | 0.0000 | 0.0000 | 0.7300 | 0.0800 | 0.0000 | 0.0000 | 0.1900 | 0.0000 | 0.0000 |
| Cairns, AU | 0.0000 | 0.0000 | 0.4167 | 0.0104 | 0.0104 | 0.0000 | 0.0000 | 0.0000 | 0.0000 | 0.0000 |
| Tahiti, FP | 0.0000 | 0.0000 | 0.0417 | 0.5833 | 0.1667 | 0.0000 | 0.0000 | 0.0000 | 0.1146 | 0.0208 |

|  |  | **AG2 locus** | |  |  |  |  |  |  |  |  |  |
| --- | --- | --- | --- | --- | --- | --- | --- | --- | --- | --- | --- | --- |
| **Population** |  | **AG2.135** | **AG2.137** | | **AG2.139** | **AG2.141** | **AG2.143** | **AG2.145** | **AG2.149** | **AG2.151** | **AG2.153** | **AG2.155** |
| Madera, California, USA |  | 0.0000 | 0.0724 | | 0.0000 | 0.0000 | 0.4342 | 0.0789 | 0.0000 | 0.0000 | 0.0000 | 0.0000 |
| Fresno, California, USA |  | 0.0000 | 0.0000 | | 0.0000 | 0.0000 | 0.0699 | 0.0000 | 0.0000 | 0.0000 | 0.0000 | 0.0000 |
| San Mateo County, California, USA |  | 0.0000 | 0.0938 | | 0.0000 | 0.0000 | 0.2813 | 0.0625 | 0.1563 | 0.0000 | 0.0000 | 0.0000 |
| Amacuzac, Morelos, MEX |  | 0.0185 | 0.0000 | | 0.0000 | 0.0000 | 0.0000 | 0.0000 | 0.0463 | 0.0185 | 0.0000 | 0.0000 |
| Tucson (TJC2), Arizona, USA |  | 0.0093 | 0.0000 | | 0.0000 | 0.0000 | 0.0000 | 0.0278 | 0.0000 | 0.0278 | 0.0463 | 0.0000 |
| Mazatan, Chiapas, MEX |  | 0.0111 | 0.0000 | | 0.0000 | 0.0000 | 0.0000 | 0.0000 | 0.0000 | 0.0000 | 0.0000 | 0.0000 |
| Muscogee County, Georgia, USA |  | 0.0091 | 0.0000 | | 0.0000 | 0.0000 | 0.0636 | 0.0636 | 0.0182 | 0.0000 | 0.0000 | 0.0000 |
| Tucson, Arizona, USA |  | 0.0000 | 0.0000 | | 0.0000 | 0.0000 | 0.0000 | 0.0185 | 0.0093 | 0.0093 | 0.0648 | 0.0370 |
| Tapachula, Chiapas, MEX |  | 0.0463 | 0.0000 | | 0.0000 | 0.0000 | 0.0000 | 0.0000 | 0.0000 | 0.0000 | 0.0000 | 0.0000 |
| Maricopa County, Arizona, USA |  | 0.0000 | 0.0000 | | 0.0094 | 0.0000 | 0.0000 | 0.0094 | 0.0000 | 0.0283 | 0.0189 | 0.0000 |
| Hermosillo, Sonora, MEX |  | 0.0000 | 0.0000 | | 0.0000 | 0.0000 | 0.0500 | 0.0500 | 0.0600 | 0.0100 | 0.0100 | 0.0000 |
| Nogales, Sonora, MEX |  | 0.0000 | 0.0000 | | 0.0000 | 0.0000 | 0.0000 | 0.0686 | 0.0392 | 0.0980 | 0.0196 | 0.0196 |
| Tijuana, Baja California Norte, MEX |  | 0.0000 | 0.2500 | | 0.0000 | 0.0000 | 0.0000 | 0.4500 | 0.0000 | 0.2250 | 0.0000 | 0.0000 |
| Houston 2009, Texas, USA |  | 0.0000 | 0.0000 | | 0.0000 | 0.0000 | 0.0000 | 0.0000 | 0.0000 | 0.0000 | 0.0000 | 0.0000 |
| Houston 2011, Texas, USA |  | 0.0000 | 0.0000 | | 0.0000 | 0.0000 | 0.0000 | 0.0000 | 0.0000 | 0.0000 | 0.0000 | 0.0000 |
| New Orleans, New Orleans, USA |  | 0.0000 | 0.0000 | | 0.0000 | 0.0111 | 0.0444 | 0.0111 | 0.0111 | 0.0000 | 0.0000 | 0.0000 |
| Miami, Florida, USA |  | 0.0000 | 0.0000 | | 0.0116 | 0.0000 | 0.0465 | 0.0116 | 0.0000 | 0.1047 | 0.0000 | 0.0000 |
| Vaca Key, Florida, USA |  | 0.0357 | 0.0000 | | 0.0357 | 0.2024 | 0.0000 | 0.0000 | 0.0000 | 0.0476 | 0.0357 | 0.0000 |
| Pijijiapan, Chiapas, MEX |  | 0.0532 | 0.0000 | | 0.0000 | 0.0000 | 0.0000 | 0.0000 | 0.0000 | 0.0000 | 0.0000 | 0.0000 |
| Coatzacoalcos, Veracruz, MEX |  | 0.3200 | 0.0000 | | 0.0000 | 0.0000 | 0.0000 | 0.0000 | 0.0000 | 0.0000 | 0.0000 | 0.0000 |
| Puerto Rico |  | 0.1887 | 0.0472 | | 0.0472 | 0.0094 | 0.0000 | 0.0000 | 0.0000 | 0.0000 | 0.0000 | 0.0000 |
| Cali, COL |  | 0.0000 | 0.0000 | | 0.0063 | 0.0938 | 0.0750 | 0.0250 | 0.0000 | 0.0000 | 0.0000 | 0.0250 |
| Cachoeiro, BRA |  | 0.0000 | 0.0000 | | 0.0000 | 0.0000 | 0.0217 | 0.0072 | 0.0000 | 0.0000 | 0.0000 | 0.0072 |
| Maraba, BRA |  | 0.1146 | 0.0417 | | 0.0000 | 0.0000 | 0.0208 | 0.0208 | 0.0000 | 0.0000 | 0.0104 | 0.0208 |
| Natal, BRA |  | 0.0213 | 0.0000 | | 0.0000 | 0.0000 | 0.0106 | 0.0000 | 0.0000 | 0.0000 | 0.0213 | 0.1064 |
| Jacobina, BRA |  | 0.0000 | 0.0000 | | 0.0000 | 0.0000 | 0.0106 | 0.0213 | 0.0000 | 0.0000 | 0.0000 | 0.0053 |
| Bolivar, VEN |  | 0.0000 | 0.0104 | | 0.0000 | 0.0000 | 0.0104 | 0.0104 | 0.0000 | 0.0729 | 0.0000 | 0.0521 |
| Dominica, DOM |  | 0.0053 | 0.0000 | | 0.0947 | 0.0579 | 0.0316 | 0.0000 | 0.0000 | 0.0000 | 0.0000 | 0.0000 |
| Jeddah, SA |  | 0.0061 | 0.0061 | | 0.0000 | 0.0000 | 0.0061 | 0.0061 | 0.0183 | 0.0671 | 0.0732 | 0.0549 |
| Prachuabkhirikan, THA |  | 0.0319 | 0.0213 | | 0.0000 | 0.0213 | 0.0000 | 0.0000 | 0.0106 | 0.0000 | 0.0000 | 0.0000 |
| Bangkok, THA |  | 0.0000 | 0.0000 | | 0.0000 | 0.0000 | 0.0000 | 0.0000 | 0.0000 | 0.0000 | 0.0000 | 0.0000 |
| Cairns, AU |  | 0.0000 | 0.0000 | | 0.0000 | 0.0000 | 0.0000 | 0.0000 | 0.0000 | 0.0000 | 0.0000 | 0.0000 |
| Tahiti, FP |  | 0.0208 | 0.0000 | | 0.0000 | 0.0000 | 0.0000 | 0.0000 | 0.0313 | 0.0000 | 0.0000 | 0.0104 |

|  |  | **AG2 locus** | |  |  |  |  |  |  |  |  |
| --- | --- | --- | --- | --- | --- | --- | --- | --- | --- | --- | --- |
| **Population** |  | **AG2.157** | **AG2.159** | | **AG2.161** | **AG2.163** | **AG2.165** | **AG2.167** | **AG2.169** | **AG2.171** | **AG2.173** |
| Madera, California, USA |  | 0.0000 | 0.0000 | | 0.0000 | 0.0000 | 0.0000 | 0.0000 | 0.0000 | 0.0000 | 0.0000 |
| Fresno, California, USA |  | 0.0000 | 0.0000 | | 0.0000 | 0.0000 | 0.0000 | 0.0000 | 0.0000 | 0.0000 | 0.0000 |
| San Mateo County, California, USA |  | 0.0000 | 0.0000 | | 0.0000 | 0.0000 | 0.0000 | 0.0000 | 0.0000 | 0.0000 | 0.0000 |
| Amacuzac, Morelos, MEX |  | 0.0000 | 0.0000 | | 0.0000 | 0.0000 | 0.0000 | 0.0000 | 0.0000 | 0.0000 | 0.0000 |
| Tucson (TJC2), Arizona, USA |  | 0.0000 | 0.0000 | | 0.0000 | 0.0000 | 0.0000 | 0.0000 | 0.0000 | 0.0000 | 0.0000 |
| Mazatan, Chiapas, MEX |  | 0.0000 | 0.0000 | | 0.0000 | 0.0000 | 0.0000 | 0.0000 | 0.0000 | 0.0000 | 0.0000 |
| Muscogee County, Georgia, USA |  | 0.0000 | 0.0091 | | 0.0091 | 0.0000 | 0.0000 | 0.0000 | 0.0000 | 0.0000 | 0.0000 |
| Tucson, Arizona, USA |  | 0.0741 | 0.0000 | | 0.0000 | 0.0000 | 0.0000 | 0.0000 | 0.0000 | 0.0000 | 0.0000 |
| Tapachula, Chiapas, MEX |  | 0.0000 | 0.0000 | | 0.0000 | 0.0000 | 0.0000 | 0.0000 | 0.0000 | 0.0000 | 0.0000 |
| Maricopa County, Arizona, USA |  | 0.0000 | 0.0000 | | 0.0000 | 0.0000 | 0.0000 | 0.0000 | 0.0000 | 0.0000 | 0.0000 |
| Hermosillo, Sonora, MEX |  | 0.0000 | 0.0000 | | 0.0000 | 0.0000 | 0.0000 | 0.0000 | 0.0000 | 0.0000 | 0.0000 |
| Nogales, Sonora, MEX |  | 0.0000 | 0.0000 | | 0.0000 | 0.0000 | 0.0000 | 0.0000 | 0.0000 | 0.0000 | 0.0000 |
| Tijuana, Baja California Norte, MEX |  | 0.0000 | 0.0000 | | 0.0000 | 0.0000 | 0.0000 | 0.0000 | 0.0000 | 0.0000 | 0.0000 |
| Houston 2009, Texas, USA |  | 0.0000 | 0.0000 | | 0.0000 | 0.0000 | 0.0000 | 0.0000 | 0.0000 | 0.0000 | 0.0000 |
| Houston 2011, Texas, USA |  | 0.0000 | 0.0000 | | 0.0000 | 0.0000 | 0.0000 | 0.0000 | 0.0000 | 0.0000 | 0.0000 |
| New Orleans, New Orleans, USA |  | 0.0000 | 0.0000 | | 0.0000 | 0.0000 | 0.0000 | 0.0000 | 0.0000 | 0.0000 | 0.0000 |
| Miami, Florida, USA |  | 0.0000 | 0.0000 | | 0.0000 | 0.0000 | 0.0000 | 0.0000 | 0.0000 | 0.0000 | 0.0000 |
| Vaca Key, Florida, USA |  | 0.0000 | 0.0000 | | 0.0000 | 0.0000 | 0.0000 | 0.0000 | 0.0000 | 0.0000 | 0.0000 |
| Pijijiapan, Chiapas, MEX |  | 0.0000 | 0.0000 | | 0.0000 | 0.0000 | 0.0000 | 0.0000 | 0.0000 | 0.0000 | 0.0000 |
| Coatzacoalcos, Veracruz, MEX |  | 0.0000 | 0.0000 | | 0.0000 | 0.0000 | 0.0000 | 0.0000 | 0.0000 | 0.0000 | 0.0000 |
| Puerto Rico |  | 0.0755 | 0.0000 | | 0.0000 | 0.0000 | 0.0000 | 0.0000 | 0.0000 | 0.0000 | 0.0000 |
| Cali, COL |  | 0.0188 | 0.0000 | | 0.0063 | 0.0000 | 0.0000 | 0.0000 | 0.0000 | 0.0000 | 0.0000 |
| Cachoeiro, BRA |  | 0.1377 | 0.0870 | | 0.0000 | 0.0000 | 0.0000 | 0.0580 | 0.0435 | 0.0000 | 0.0072 |
| Maraba, BRA |  | 0.0000 | 0.0000 | | 0.0000 | 0.0000 | 0.0000 | 0.0000 | 0.0000 | 0.0000 | 0.0000 |
| Natal, BRA |  | 0.0319 | 0.0000 | | 0.0000 | 0.0000 | 0.0000 | 0.0426 | 0.1064 | 0.0213 | 0.0000 |
| Jacobina, BRA |  | 0.0372 | 0.0319 | | 0.0160 | 0.0000 | 0.0053 | 0.1277 | 0.0213 | 0.0000 | 0.0000 |
| Bolivar, VEN |  | 0.0729 | 0.0000 | | 0.0000 | 0.0000 | 0.0000 | 0.0000 | 0.0000 | 0.0000 | 0.0000 |
| Dominica, DOM |  | 0.0000 | 0.0000 | | 0.0000 | 0.0000 | 0.0000 | 0.0000 | 0.0000 | 0.0000 | 0.0000 |
| Jeddah, SA |  | 0.0061 | 0.0488 | | 0.0000 | 0.0061 | 0.0000 | 0.0061 | 0.0000 | 0.0000 | 0.0000 |
| Prachuabkhirikan, THA |  | 0.0000 | 0.0000 | | 0.0000 | 0.0000 | 0.0000 | 0.0000 | 0.0000 | 0.0000 | 0.0000 |
| Bangkok, THA |  | 0.0000 | 0.0000 | | 0.0000 | 0.0000 | 0.0000 | 0.0000 | 0.0000 | 0.0000 | 0.0000 |
| Cairns, AU |  | 0.0000 | 0.2188 | | 0.1250 | 0.0417 | 0.0000 | 0.0104 | 0.1667 | 0.0000 | 0.0000 |
| Tahiti, FP |  | 0.0000 | 0.0104 | | 0.0000 | 0.0000 | 0.0000 | 0.0000 | 0.0000 | 0.0000 | 0.0000 |

|  | **AG5 locus** |  |  |  |  |  |  |  |  |  |
| --- | --- | --- | --- | --- | --- | --- | --- | --- | --- | --- |
| **Population** | **AG5.164** | **AG5.166** | **AG5.168** | **AG5.170** | **AG5.172** | **AG5.174** | **AG5.176** | **AG5.178** | **AG5.180** | **AG5.182** |
| Madera, California, USA | 0.0000 | 0.0260 | 0.1494 | 0.0000 | 0.1169 | 0.0000 | 0.2143 | 0.4935 | 0.0000 | 0.0000 |
| Fresno, California, USA | 0.0000 | 0.2737 | 0.1000 | 0.0632 | 0.1474 | 0.0000 | 0.1053 | 0.3105 | 0.0000 | 0.0000 |
| San Mateo County, California, USA | 0.0000 | 0.0313 | 0.1563 | 0.0000 | 0.0313 | 0.0000 | 0.1875 | 0.5938 | 0.0000 | 0.0000 |
| Amacuzac, Morelos, MEX | 0.0000 | 0.1019 | 0.1204 | 0.0000 | 0.1111 | 0.0000 | 0.0556 | 0.6111 | 0.0000 | 0.0000 |
| Tucson (TJC2), Arizona, USA | 0.0000 | 0.0556 | 0.2130 | 0.0000 | 0.4907 | 0.0000 | 0.1481 | 0.0926 | 0.0000 | 0.0000 |
| Mazatan, Chiapas, MEX | 0.0000 | 0.0000 | 0.2111 | 0.0000 | 0.1111 | 0.0000 | 0.4444 | 0.2333 | 0.0000 | 0.0000 |
| Muscogee County, Georgia, USA | 0.0000 | 0.0000 | 0.2685 | 0.1759 | 0.2593 | 0.0463 | 0.0463 | 0.2037 | 0.0000 | 0.0000 |
| Tucson, Arizona, USA | 0.0000 | 0.0000 | 0.1204 | 0.0000 | 0.4074 | 0.0185 | 0.2315 | 0.2222 | 0.0000 | 0.0000 |
| Tapachula, Chiapas, MEX | 0.0000 | 0.0463 | 0.0278 | 0.0000 | 0.1389 | 0.0000 | 0.4444 | 0.3426 | 0.0000 | 0.0000 |
| Maricopa County, Arizona, USA | 0.0000 | 0.0000 | 0.2547 | 0.0566 | 0.4057 | 0.0000 | 0.1604 | 0.1226 | 0.0000 | 0.0000 |
| Hermosillo, Sonora, MEX | 0.0000 | 0.0800 | 0.0200 | 0.0000 | 0.5500 | 0.0000 | 0.0100 | 0.3400 | 0.0000 | 0.0000 |
| Nogales, Sonora, MEX | 0.0000 | 0.0196 | 0.0980 | 0.0000 | 0.7353 | 0.0000 | 0.0000 | 0.1471 | 0.0000 | 0.0000 |
| Tijuana, Baja California Norte, MEX | 0.0000 | 0.2000 | 0.0000 | 0.0000 | 0.5500 | 0.0000 | 0.0500 | 0.2000 | 0.0000 | 0.0000 |
| Houston 2009, Texas, USA | 0.0000 | 0.0000 | 0.3103 | 0.2931 | 0.1897 | 0.0000 | 0.0000 | 0.2069 | 0.0000 | 0.0000 |
| Houston 2011, Texas, USA | 0.0000 | 0.0526 | 0.1316 | 0.0000 | 0.4737 | 0.0000 | 0.0000 | 0.3421 | 0.0000 | 0.0000 |
| New Orleans, New Orleans, USA | 0.0000 | 0.0341 | 0.0795 | 0.1932 | 0.2159 | 0.0000 | 0.2273 | 0.2500 | 0.0000 | 0.0000 |
| Miami, Florida, USA | 0.0000 | 0.0532 | 0.1489 | 0.0851 | 0.2128 | 0.0319 | 0.1596 | 0.3085 | 0.0000 | 0.0000 |
| Vaca Key, Florida, USA | 0.0000 | 0.1190 | 0.1786 | 0.0119 | 0.1786 | 0.0119 | 0.2024 | 0.2976 | 0.0000 | 0.0000 |
| Pijijiapan, Chiapas, MEX | 0.0000 | 0.0000 | 0.4787 | 0.0000 | 0.0426 | 0.0000 | 0.2553 | 0.2234 | 0.0000 | 0.0000 |
| Coatzacoalcos, Veracruz, MEX | 0.0000 | 0.0000 | 0.0000 | 0.0000 | 0.0800 | 0.0000 | 0.9200 | 0.0000 | 0.0000 | 0.0000 |
| Puerto Rico | 0.0463 | 0.1852 | 0.2130 | 0.0000 | 0.0463 | 0.0741 | 0.0926 | 0.3148 | 0.0278 | 0.0000 |
| Cali, COL | 0.0000 | 0.4500 | 0.3063 | 0.0000 | 0.0188 | 0.0000 | 0.2250 | 0.0000 | 0.0000 | 0.0000 |
| Cachoeiro, BRA | 0.0000 | 0.0214 | 0.3643 | 0.0071 | 0.0429 | 0.0000 | 0.4643 | 0.1000 | 0.0000 | 0.0000 |
| Maraba, BRA | 0.0000 | 0.0729 | 0.1667 | 0.0313 | 0.0313 | 0.0000 | 0.3021 | 0.3958 | 0.0000 | 0.0000 |
| Natal, BRA | 0.0000 | 0.0106 | 0.5106 | 0.0000 | 0.0426 | 0.0000 | 0.3936 | 0.0426 | 0.0000 | 0.0000 |
| Jacobina, BRA | 0.0000 | 0.0054 | 0.4140 | 0.0000 | 0.0054 | 0.0000 | 0.4839 | 0.0914 | 0.0000 | 0.0000 |
| Bolivar, VEN | 0.0000 | 0.3854 | 0.2188 | 0.0000 | 0.0833 | 0.0000 | 0.1979 | 0.1146 | 0.0000 | 0.0000 |
| Dominica, DOM | 0.0000 | 0.1000 | 0.1947 | 0.0000 | 0.0368 | 0.1632 | 0.3842 | 0.1211 | 0.0000 | 0.0000 |
| Jeddah, SA | 0.0000 | 0.0000 | 0.1905 | 0.0952 | 0.2560 | 0.0119 | 0.0714 | 0.1786 | 0.1964 | 0.0000 |
| Prachuabkhirikan, THA | 0.0000 | 0.0000 | 0.0106 | 0.0213 | 0.0426 | 0.0000 | 0.2872 | 0.5319 | 0.0638 | 0.0106 |
| Bangkok, THA | 0.0000 | 0.0000 | 0.0000 | 0.0000 | 0.0000 | 0.0000 | 0.5900 | 0.4100 | 0.0000 | 0.0000 |
| Cairns, AU | 0.0000 | 0.0000 | 0.0000 | 0.0000 | 0.2708 | 0.0313 | 0.0000 | 0.5417 | 0.1563 | 0.0000 |
| Tahiti, FP | 0.0000 | 0.0208 | 0.0000 | 0.0000 | 0.3438 | 0.0104 | 0.0729 | 0.2708 | 0.2813 | 0.0000 |

|  |  | **AG5 locus** | |
| --- | --- | --- | --- |
| **Population** |  | **AG5.184** |  |
| Madera, California, USA |  | 0.0000 |  |
| Fresno, California, USA |  | 0.0000 |  |
| San Mateo County, California, USA |  | 0.0000 |  |
| Amacuzac, Morelos, MEX |  | 0.0000 |  |
| Tucson (TJC2), Arizona, USA |  | 0.0000 |  |
| Mazatan, Chiapas, MEX |  | 0.0000 |  |
| Muscogee County, Georgia, USA |  | 0.0000 |  |
| Tucson, Arizona, USA |  | 0.0000 |  |
| Tapachula, Chiapas, MEX |  | 0.0000 |  |
| Maricopa County, Arizona, USA |  | 0.0000 |  |
| Hermosillo, Sonora, MEX |  | 0.0000 |  |
| Nogales, Sonora, MEX |  | 0.0000 |  |
| Tijuana, Baja California Norte, MEX |  | 0.0000 |  |
| Houston 2009, Texas, USA |  | 0.0000 |  |
| Houston 2011, Texas, USA |  | 0.0000 |  |
| New Orleans, New Orleans, USA |  | 0.0000 |  |
| Miami, Florida, USA |  | 0.0000 |  |
| Vaca Key, Florida, USA |  | 0.0000 |  |
| Pijijiapan, Chiapas, MEX |  | 0.0000 |  |
| Coatzacoalcos, Veracruz, MEX |  | 0.0000 |  |
| Puerto Rico |  | 0.0000 |  |
| Cali, COL |  | 0.0000 |  |
| Cachoeiro, BRA |  | 0.0000 |  |
| Maraba, BRA |  | 0.0000 |  |
| Natal, BRA |  | 0.0000 |  |
| Jacobina, BRA |  | 0.0000 |  |
| Bolivar, VEN |  | 0.0000 |  |
| Dominica, DOM |  | 0.0000 |  |
| Jeddah, SA |  | 0.0000 |  |
| Prachuabkhirikan, THA |  | 0.0319 |  |
| Bangkok, THA |  | 0.0000 |  |
| Cairns, AU |  | 0.0000 |  |
| Tahiti, FP |  | 0.0000 |  |

|  | **A1 locus** |  |  |  |  |  |  |  |  |  |
| --- | --- | --- | --- | --- | --- | --- | --- | --- | --- | --- |
| **Population** | **A1.149** | **A1.156** | **A1.157** | **A1.158** | **A1.159** | **A1.162** | **A1.165** | **A1.168** | **A1.171** | **A1.174** |
| Madera, California, USA | 0.0000 | 0.0000 | 0.0000 | 0.3636 | 0.0000 | 0.4286 | 0.0000 | 0.2078 | 0.0000 | 0.0000 |
| Fresno, California, USA | 0.0000 | 0.0000 | 0.0000 | 0.5638 | 0.0000 | 0.0213 | 0.0000 | 0.2021 | 0.2128 | 0.0000 |
| San Mateo County, California, USA | 0.0000 | 0.0000 | 0.0000 | 0.5625 | 0.0000 | 0.1250 | 0.0000 | 0.2500 | 0.0625 | 0.0000 |
| Amacuzac, Morelos, MEX | 0.0000 | 0.0000 | 0.0000 | 0.3704 | 0.0000 | 0.1574 | 0.0000 | 0.4352 | 0.0370 | 0.0000 |
| Tucson (TJC2), Arizona, USA | 0.0000 | 0.0000 | 0.0000 | 0.2593 | 0.0000 | 0.0185 | 0.0000 | 0.5648 | 0.1574 | 0.0000 |
| Mazatan, Chiapas, MEX | 0.0000 | 0.0000 | 0.0000 | 0.4000 | 0.0556 | 0.0000 | 0.0000 | 0.1667 | 0.3778 | 0.0000 |
| Muscogee County, Georgia, USA | 0.0000 | 0.0000 | 0.0000 | 0.2545 | 0.0455 | 0.1909 | 0.0000 | 0.1364 | 0.3727 | 0.0000 |
| Tucson, Arizona, USA | 0.0000 | 0.0093 | 0.0000 | 0.2870 | 0.0000 | 0.1111 | 0.0000 | 0.4444 | 0.1481 | 0.0000 |
| Tapachula, Chiapas, MEX | 0.0000 | 0.0000 | 0.0000 | 0.5926 | 0.0278 | 0.0000 | 0.0000 | 0.0278 | 0.3519 | 0.0000 |
| Maricopa County, Arizona, USA | 0.0000 | 0.0000 | 0.0000 | 0.1698 | 0.0000 | 0.1132 | 0.0000 | 0.6132 | 0.1038 | 0.0000 |
| Hermosillo, Sonora, MEX | 0.0000 | 0.0000 | 0.0000 | 0.2200 | 0.0000 | 0.0000 | 0.0100 | 0.6200 | 0.1500 | 0.0000 |
| Nogales, Sonora, MEX | 0.0000 | 0.0000 | 0.0000 | 0.1078 | 0.0000 | 0.0000 | 0.0000 | 0.7255 | 0.1471 | 0.0196 |
| Tijuana, Baja California Norte, MEX | 0.0000 | 0.0000 | 0.0000 | 0.2750 | 0.0000 | 0.0000 | 0.0000 | 0.2750 | 0.4500 | 0.0000 |
| Houston 2009, Texas, USA | 0.0000 | 0.0000 | 0.0000 | 0.6034 | 0.0000 | 0.3103 | 0.0000 | 0.0000 | 0.0862 | 0.0000 |
| Houston 2011, Texas, USA | 0.0000 | 0.0000 | 0.0000 | 0.1579 | 0.0000 | 0.0263 | 0.0000 | 0.5263 | 0.2895 | 0.0000 |
| New Orleans, New Orleans, USA | 0.0000 | 0.0455 | 0.0000 | 0.2273 | 0.0568 | 0.1023 | 0.0000 | 0.3409 | 0.2273 | 0.0000 |
| Miami, Florida, USA | 0.0000 | 0.1596 | 0.0000 | 0.2340 | 0.1489 | 0.0213 | 0.0000 | 0.1702 | 0.2660 | 0.0000 |
| Vaca Key, Florida, USA | 0.0119 | 0.0357 | 0.0000 | 0.2857 | 0.1190 | 0.0119 | 0.0000 | 0.2381 | 0.2976 | 0.0000 |
| Pijijiapan, Chiapas, MEX | 0.0000 | 0.0000 | 0.0000 | 0.4787 | 0.2128 | 0.0532 | 0.0000 | 0.0851 | 0.1702 | 0.0000 |
| Coatzacoalcos, Veracruz, MEX | 0.0000 | 0.3100 | 0.0000 | 0.1800 | 0.0000 | 0.0000 | 0.0000 | 0.2000 | 0.3100 | 0.0000 |
| Puerto Rico | 0.0000 | 0.0000 | 0.0000 | 0.1574 | 0.0556 | 0.1667 | 0.0000 | 0.4722 | 0.1481 | 0.0000 |
| Cali, COL | 0.0000 | 0.0000 | 0.0063 | 0.0813 | 0.0000 | 0.0500 | 0.0000 | 0.4688 | 0.3938 | 0.0000 |
| Cachoeiro, BRA | 0.0000 | 0.0571 | 0.0000 | 0.0429 | 0.0000 | 0.1000 | 0.0000 | 0.8000 | 0.0000 | 0.0000 |
| Maraba, BRA | 0.0000 | 0.1146 | 0.0000 | 0.0000 | 0.0000 | 0.2917 | 0.0000 | 0.5833 | 0.0104 | 0.0000 |
| Natal, BRA | 0.0000 | 0.0851 | 0.0000 | 0.0000 | 0.0000 | 0.2234 | 0.0000 | 0.6702 | 0.0213 | 0.0000 |
| Jacobina, BRA | 0.0000 | 0.0957 | 0.0000 | 0.0372 | 0.0000 | 0.1862 | 0.0000 | 0.6809 | 0.0000 | 0.0000 |
| Bolivar, VEN | 0.0000 | 0.0000 | 0.0000 | 0.3854 | 0.0000 | 0.5313 | 0.0000 | 0.0729 | 0.0104 | 0.0000 |
| Dominica, DOM | 0.0000 | 0.0000 | 0.0000 | 0.1000 | 0.0000 | 0.2421 | 0.0000 | 0.6579 | 0.0000 | 0.0000 |
| Jeddah, SA | 0.0000 | 0.0476 | 0.0000 | 0.3690 | 0.0000 | 0.4405 | 0.0000 | 0.1429 | 0.0000 | 0.0000 |
| Prachuabkhirikan, THA | 0.0000 | 0.0426 | 0.0000 | 0.0851 | 0.0000 | 0.1915 | 0.0000 | 0.6170 | 0.0638 | 0.0000 |
| Bangkok, THA | 0.0000 | 0.0000 | 0.0000 | 0.0000 | 0.0000 | 0.1800 | 0.0000 | 0.8200 | 0.0000 | 0.0000 |
| Cairns, AU | 0.0000 | 0.0000 | 0.0000 | 0.0313 | 0.0833 | 0.2292 | 0.0000 | 0.6563 | 0.0000 | 0.0000 |
| Tahiti, FP | 0.0000 | 0.0000 | 0.0000 | 0.1042 | 0.0729 | 0.2396 | 0.0000 | 0.5417 | 0.0417 | 0.0000 |

|  | **A9 locus** |  |  |  |  |  |  |  |
| --- | --- | --- | --- | --- | --- | --- | --- | --- |
| **Population** | **A9.181** | **A9.182** | **A9.188** | **A9.189** | **A9.190** | **A9.191** | **A9.194** | **A9.197** |
| Madera, California, USA | 0.0000 | 0.5130 | 0.0974 | 0.0000 | 0.0000 | 0.2857 | 0.1039 | 0.0000 |
| Fresno, California, USA | 0.0000 | 0.5165 | 0.2363 | 0.0000 | 0.0000 | 0.1868 | 0.0604 | 0.0000 |
| San Mateo County, California, USA | 0.0000 | 0.4333 | 0.0667 | 0.0000 | 0.0000 | 0.3000 | 0.2000 | 0.0000 |
| Amacuzac, Morelos, MEX | 0.0000 | 0.4167 | 0.0556 | 0.0000 | 0.0000 | 0.5278 | 0.0000 | 0.0000 |
| Tucson (TJC2), Arizona, USA | 0.0000 | 0.4537 | 0.4444 | 0.0000 | 0.0185 | 0.0833 | 0.0000 | 0.0000 |
| Mazatan, Chiapas, MEX | 0.0000 | 0.2222 | 0.6444 | 0.0000 | 0.0000 | 0.1333 | 0.0000 | 0.0000 |
| Muscogee County, Georgia, USA | 0.0000 | 0.1667 | 0.2870 | 0.0000 | 0.0000 | 0.5370 | 0.0093 | 0.0000 |
| Tucson, Arizona, USA | 0.0000 | 0.6389 | 0.1944 | 0.0000 | 0.0000 | 0.1667 | 0.0000 | 0.0000 |
| Tapachula, Chiapas, MEX | 0.0000 | 0.1944 | 0.6019 | 0.0000 | 0.0000 | 0.2037 | 0.0000 | 0.0000 |
| Maricopa County, Arizona, USA | 0.0000 | 0.7404 | 0.1250 | 0.0000 | 0.0000 | 0.1346 | 0.0000 | 0.0000 |
| Hermosillo, Sonora, MEX | 0.0000 | 0.7347 | 0.1020 | 0.0000 | 0.0000 | 0.1020 | 0.0612 | 0.0000 |
| Nogales, Sonora, MEX | 0.0000 | 0.5978 | 0.2826 | 0.0000 | 0.0000 | 0.1087 | 0.0109 | 0.0000 |
| Tijuana, Baja California Norte, MEX | 0.0000 | 0.7250 | 0.2000 | 0.0000 | 0.0000 | 0.0750 | 0.0000 | 0.0000 |
| Houston 2009, Texas, USA | 0.0000 | 0.7414 | 0.0345 | 0.0000 | 0.0000 | 0.2241 | 0.0000 | 0.0000 |
| Houston 2011, Texas, USA | 0.0000 | 0.9474 | 0.0263 | 0.0000 | 0.0000 | 0.0263 | 0.0000 | 0.0000 |
| New Orleans, New Orleans, USA | 0.0217 | 0.2391 | 0.2391 | 0.0000 | 0.0000 | 0.4022 | 0.0978 | 0.0000 |
| Miami, Florida, USA | 0.0000 | 0.3511 | 0.1702 | 0.0000 | 0.0000 | 0.4362 | 0.0426 | 0.0000 |
| Vaca Key, Florida, USA | 0.0000 | 0.6071 | 0.1667 | 0.0000 | 0.0000 | 0.1786 | 0.0476 | 0.0000 |
| Pijijiapan, Chiapas, MEX | 0.0000 | 0.2234 | 0.4149 | 0.0000 | 0.0000 | 0.3617 | 0.0000 | 0.0000 |
| Coatzacoalcos, Veracruz, MEX | 0.0000 | 0.0700 | 0.9300 | 0.0000 | 0.0000 | 0.0000 | 0.0000 | 0.0000 |
| Puerto Rico | 0.0000 | 0.2963 | 0.1667 | 0.0000 | 0.0000 | 0.4074 | 0.1296 | 0.0000 |
| Cali, COL | 0.0000 | 0.3313 | 0.0063 | 0.0000 | 0.0000 | 0.6438 | 0.0188 | 0.0000 |
| Cachoeiro, BRA | 0.0000 | 0.0224 | 0.5448 | 0.0000 | 0.0000 | 0.4254 | 0.0075 | 0.0000 |
| Maraba, BRA | 0.0000 | 0.0521 | 0.4688 | 0.0000 | 0.0000 | 0.3958 | 0.0833 | 0.0000 |
| Natal, BRA | 0.0000 | 0.3617 | 0.4894 | 0.0000 | 0.0000 | 0.1489 | 0.0000 | 0.0000 |
| Jacobina, BRA | 0.0000 | 0.2926 | 0.4043 | 0.0000 | 0.0000 | 0.3032 | 0.0000 | 0.0000 |
| Bolivar, VEN | 0.0000 | 0.0000 | 0.3542 | 0.0000 | 0.0000 | 0.6458 | 0.0000 | 0.0000 |
| Dominica, DOM | 0.0000 | 0.5579 | 0.1211 | 0.0895 | 0.0000 | 0.2316 | 0.0000 | 0.0000 |
| Jeddah, SA | 0.0000 | 0.1250 | 0.0179 | 0.0000 | 0.0000 | 0.8095 | 0.0357 | 0.0119 |
| Prachuabkhirikan, THA | 0.0000 | 0.0217 | 0.2500 | 0.0000 | 0.0000 | 0.5435 | 0.1848 | 0.0000 |
| Bangkok, THA | 0.0000 | 0.0000 | 0.1400 | 0.0000 | 0.0000 | 0.7900 | 0.0700 | 0.0000 |
| Cairns, AU | 0.0000 | 0.0104 | 0.6458 | 0.0000 | 0.0000 | 0.2917 | 0.0521 | 0.0000 |
| Tahiti, FP | 0.0000 | 0.1458 | 0.1354 | 0.0000 | 0.0000 | 0.6250 | 0.0938 | 0.0000 |

|  | **B2 locus** |  |  |  |  |  |  |
| --- | --- | --- | --- | --- | --- | --- | --- |
| **Population** | **B2.009** | **B2.095** | **B2.101** | **B2.104** | **B2.107** | **B2.113** | **B2.595** |
| Madera, California, USA | 0.0000 | 0.0195 | 0.0000 | 0.0000 | 0.0065 | 0.9740 | 0.0000 |
| Fresno, California, USA | 0.0000 | 0.0684 | 0.0000 | 0.0000 | 0.0000 | 0.9316 | 0.0000 |
| San Mateo County, California, USA | 0.0000 | 0.0000 | 0.0000 | 0.0000 | 0.0938 | 0.9063 | 0.0000 |
| Amacuzac, Morelos, MEX | 0.0000 | 0.0648 | 0.0000 | 0.0000 | 0.0833 | 0.8519 | 0.0000 |
| Tucson (TJC2), Arizona, USA | 0.0185 | 0.0648 | 0.0000 | 0.1667 | 0.1574 | 0.5741 | 0.0185 |
| Mazatan, Chiapas, MEX | 0.0000 | 0.0000 | 0.0000 | 0.0000 | 0.0444 | 0.9556 | 0.0000 |
| Muscogee County, Georgia, USA | 0.0091 | 0.0909 | 0.0000 | 0.0000 | 0.0273 | 0.8636 | 0.0091 |
| Tucson, Arizona, USA | 0.0185 | 0.0185 | 0.0000 | 0.2222 | 0.0648 | 0.6574 | 0.0185 |
| Tapachula, Chiapas, MEX | 0.0000 | 0.0000 | 0.0000 | 0.0000 | 0.0463 | 0.9537 | 0.0000 |
| Maricopa County, Arizona, USA | 0.0189 | 0.0849 | 0.0000 | 0.2170 | 0.0283 | 0.6321 | 0.0189 |
| Hermosillo, Sonora, MEX | 0.0000 | 0.1400 | 0.0000 | 0.4200 | 0.0700 | 0.3700 | 0.0000 |
| Nogales, Sonora, MEX | 0.0098 | 0.1373 | 0.0000 | 0.2255 | 0.1176 | 0.5000 | 0.0098 |
| Tijuana, Baja California Norte, MEX | 0.0000 | 0.0000 | 0.0000 | 0.0000 | 0.1750 | 0.8250 | 0.0000 |
| Houston 2009, Texas, USA | 0.0000 | 0.0000 | 0.0000 | 0.0000 | 0.0172 | 0.9828 | 0.0000 |
| Houston 2011, Texas, USA | 0.0000 | 0.0000 | 0.0000 | 0.0000 | 0.0000 | 1.0000 | 0.0000 |
| New Orleans, New Orleans, USA | 0.0000 | 0.1522 | 0.0000 | 0.0109 | 0.0870 | 0.7500 | 0.0000 |
| Miami, Florida, USA | 0.0000 | 0.1170 | 0.0000 | 0.0000 | 0.0000 | 0.8830 | 0.0000 |
| Vaca Key, Florida, USA | 0.0000 | 0.1429 | 0.0000 | 0.0000 | 0.0000 | 0.8571 | 0.0000 |
| Pijijiapan, Chiapas, MEX | 0.0000 | 0.0000 | 0.0000 | 0.0000 | 0.0213 | 0.9787 | 0.0000 |
| Coatzacoalcos, Veracruz, MEX | 0.0000 | 0.0000 | 0.0000 | 0.0000 | 0.0000 | 1.0000 | 0.0000 |
| Puerto Rico | 0.0000 | 0.0000 | 0.0000 | 0.0000 | 0.0463 | 0.9537 | 0.0000 |
| Cali, COL | 0.0125 | 0.0438 | 0.0000 | 0.0000 | 0.0000 | 0.9313 | 0.0125 |
| Cachoeiro, BRA | 0.0071 | 0.1286 | 0.0000 | 0.0000 | 0.0000 | 0.8571 | 0.0071 |
| Maraba, BRA | 0.0104 | 0.0938 | 0.0000 | 0.0000 | 0.0000 | 0.8854 | 0.0104 |
| Natal, BRA | 0.0106 | 0.0957 | 0.0000 | 0.0000 | 0.0000 | 0.8830 | 0.0106 |
| Jacobina, BRA | 0.0160 | 0.1170 | 0.0000 | 0.0000 | 0.0053 | 0.8457 | 0.0160 |
| Bolivar, VEN | 0.0000 | 0.0000 | 0.0000 | 0.0000 | 0.4479 | 0.5521 | 0.0000 |
| Dominica, DOM | 0.0000 | 0.0000 | 0.0000 | 0.0000 | 0.0000 | 1.0000 | 0.0000 |
| Jeddah, SA | 0.0119 | 0.0774 | 0.0000 | 0.0000 | 0.0655 | 0.8333 | 0.0119 |
| Prachuabkhirikan, THA | 0.0000 | 0.0319 | 0.0000 | 0.0000 | 0.3298 | 0.6383 | 0.0000 |
| Bangkok, THA | 0.0000 | 0.0000 | 0.0000 | 0.0000 | 0.0000 | 1.0000 | 0.0000 |
| Cairns, AU | 0.0319 | 0.2872 | 0.0106 | 0.0000 | 0.0000 | 0.6383 | 0.0319 |
| Tahiti, FP | 0.0104 | 0.0625 | 0.2917 | 0.0000 | 0.0000 | 0.6250 | 0.0104 |

|  | **B3 locus** |  |  |  |  |  |  |
| --- | --- | --- | --- | --- | --- | --- | --- |
| **Population** | **B3.148** | **B3.160** | **B3.164** | **B3.166** | **B3.172** | **B3.175** | **B3.178** |
| Madera, California, USA | 0.1429 | 0.0000 | 0.0000 | 0.2792 | 0.1234 | 0.4545 | 0.0000 |
| Fresno, California, USA | 0.0000 | 0.0000 | 0.0000 | 0.3316 | 0.3263 | 0.3421 | 0.0000 |
| San Mateo County, California, USA | 0.0625 | 0.0000 | 0.0000 | 0.1563 | 0.0625 | 0.7188 | 0.0000 |
| Amacuzac, Morelos, MEX | 0.1019 | 0.0000 | 0.0000 | 0.2037 | 0.2685 | 0.4167 | 0.0093 |
| Tucson (TJC2), Arizona, USA | 0.1852 | 0.0000 | 0.0000 | 0.3519 | 0.1481 | 0.3148 | 0.0000 |
| Mazatan, Chiapas, MEX | 0.0667 | 0.0000 | 0.0000 | 0.7222 | 0.1000 | 0.1111 | 0.0000 |
| Muscogee County, Georgia, USA | 0.0182 | 0.0000 | 0.0000 | 0.3818 | 0.1273 | 0.4727 | 0.0000 |
| Tucson, Arizona, USA | 0.4074 | 0.0000 | 0.0000 | 0.3056 | 0.0278 | 0.2593 | 0.0000 |
| Tapachula, Chiapas, MEX | 0.0556 | 0.0000 | 0.0000 | 0.5648 | 0.2315 | 0.1481 | 0.0000 |
| Maricopa County, Arizona, USA | 0.1604 | 0.0000 | 0.0000 | 0.5472 | 0.1321 | 0.1604 | 0.0000 |
| Hermosillo, Sonora, MEX | 0.0500 | 0.0000 | 0.0000 | 0.5200 | 0.0600 | 0.3700 | 0.0000 |
| Nogales, Sonora, MEX | 0.0490 | 0.0000 | 0.0000 | 0.5196 | 0.0784 | 0.3529 | 0.0000 |
| Tijuana, Baja California Norte, MEX | 0.0000 | 0.0000 | 0.0000 | 0.6000 | 0.0000 | 0.4000 | 0.0000 |
| Houston 2009, Texas, USA | 0.0000 | 0.0000 | 0.0000 | 0.5172 | 0.1724 | 0.3103 | 0.0000 |
| Houston 2011, Texas, USA | 0.0000 | 0.0000 | 0.0000 | 0.6053 | 0.1579 | 0.2368 | 0.0000 |
| New Orleans, New Orleans, USA | 0.0000 | 0.0000 | 0.0000 | 0.6522 | 0.0761 | 0.2717 | 0.0000 |
| Miami, Florida, USA | 0.0106 | 0.0000 | 0.0000 | 0.5319 | 0.3404 | 0.1170 | 0.0000 |
| Vaca Key, Florida, USA | 0.0000 | 0.0238 | 0.0000 | 0.3214 | 0.5357 | 0.1190 | 0.0000 |
| Pijijiapan, Chiapas, MEX | 0.1383 | 0.0000 | 0.0000 | 0.7447 | 0.0851 | 0.0319 | 0.0000 |
| Coatzacoalcos, Veracruz, MEX | 0.0000 | 0.0000 | 0.0000 | 0.0900 | 0.8600 | 0.0500 | 0.0000 |
| Puerto Rico | 0.0741 | 0.0000 | 0.0000 | 0.4722 | 0.2500 | 0.2037 | 0.0000 |
| Cali, COL | 0.0000 | 0.0000 | 0.0000 | 0.7438 | 0.1000 | 0.1563 | 0.0000 |
| Cachoeiro, BRA | 0.0000 | 0.0000 | 0.0000 | 0.8071 | 0.0786 | 0.1143 | 0.0000 |
| Maraba, BRA | 0.0000 | 0.0000 | 0.0000 | 0.6146 | 0.3750 | 0.0104 | 0.0000 |
| Natal, BRA | 0.0000 | 0.0000 | 0.0000 | 0.7872 | 0.0957 | 0.1170 | 0.0000 |
| Jacobina, BRA | 0.0319 | 0.0000 | 0.0000 | 0.8085 | 0.0904 | 0.0691 | 0.0000 |
| Bolivar, VEN | 0.0208 | 0.0000 | 0.0000 | 0.7396 | 0.0833 | 0.1563 | 0.0000 |
| Dominica, DOM | 0.2474 | 0.0000 | 0.0000 | 0.1737 | 0.1158 | 0.4632 | 0.0000 |
| Jeddah, SA | 0.0357 | 0.0000 | 0.0000 | 0.6012 | 0.1190 | 0.2440 | 0.0000 |
| Prachuabkhirikan, THA | 0.1383 | 0.0000 | 0.0000 | 0.5957 | 0.2128 | 0.0532 | 0.0000 |
| Bangkok, THA | 0.4700 | 0.0000 | 0.0000 | 0.3500 | 0.1800 | 0.0000 | 0.0000 |
| Cairns, AU | 0.2188 | 0.0000 | 0.0104 | 0.2396 | 0.0000 | 0.5313 | 0.0000 |
| Tahiti, FP | 0.0000 | 0.0000 | 0.0000 | 0.6458 | 0.1563 | 0.1979 | 0.0000 |
