# Supplementary material for: Origin of the Dengue Fever Mosquito, Aedes aegypti, in California
Source: PLoS Negl Trop Dis. 2014 Jul 31;8(7):e3029. doi: 10.1371/journal.pntd.0003029 (PMC4117443; doi:10.1371/journal.pntd.0003029)
Supplement: Text S1 — Marker validation. (DOCX) [file pntd.0003029.s007.docx]

**Text S1:** Marker validation.

The 12 microsatellite markers used in this study have been previously validated in Brown et al. (*6*). Consistent with expectations, most of the loci were in HWE after Bonferroni correction (F_is_ : 92.2% and G_is_ : 94.7%). From the 33 population-specific tests performed for each marker, 0, 1, or 2 of the tests were significant for each of the loci after Bonferroni correction. Exceptions were the A9 locus, where 9 of the 33 tests were significant using both the F_is_ and G_is_ statistics, and the AG2 locus with 6 of 33 tests showing significance according to the F_is_. An excess of homozygotes has been previously reported for locus A9 and appears to be a consequence of the presence of null alleles [*6*], with no effects in the outcome of downstream population genetic analyses. A total of 90 out of the 2178 locus-by-locus tests (4.13%) were significant for LD after Bonferroni correction, with no two loci consistently linked across populations.
